# Understanding Appropriation of Digital Self-Monitoring Tools in Mental Health Care: Qualitative Analysis

**DOI:** 10.2196/60096

**Published:** 2025-03-03

**Authors:** Lena de Thurah, Glenn Kiekens, Jeroen Weermeijer, Lotte Uyttebroek, Martien Wampers, Rafaël Bonnier, Inez Myin-Germeys

**Affiliations:** 1 Department of Neurosciences, Center for Contextual Psychiatry, KU Leuven, Herestraat 49 ON5B, bus 1029, Leuven, 3000, Belgium, 32 16 32 14 57; 2 Research Unit of Clinical Psychology, Faculty of Psychology and Educational Sciences, KU Leuven, Leuven, Belgium; 3 Department of Medical and Clinical Psychology, Tilburg University, Tilburg, Netherlands

**Keywords:** digital self-monitoring, technology appropriation, experience sampling method, mental health care, mental health, self-monitoring, digital health, adoption, implementation, thematic, usability, interview, experience, attitude, opinion, perception, perspective, acceptance

## Abstract

**Background:**

Digital self-monitoring tools, such as the experience sampling method (ESM), enable individuals to collect detailed information about their mental health and daily life context and may help guide and support person-centered mental health care. However, similar to many digital interventions, the ESM struggles to move from research to clinical integration. To guide the implementation of self-monitoring tools in mental health care, it is important to understand why and how clinicians and clients adopted, adapted, and incorporated these tools in practice.

**Objective:**

Therefore, this study examined how clinicians and clients within a psychiatric center appropriated an ESM-based self-monitoring tool within their therapy.

**Methods:**

Twelve clinicians and 24 clients participated in the piloting of the ESM tool, IMPROVE. After utilizing the tool, 7 clinicians and 11 clients took part in semistructured interviews. A thematic framework analysis was performed focusing on participants’ prior knowledge and expectations, actual use in practice, and potential future use of ESM tools.

**Results:**

Many participants experienced that the ESM tool provided useful information about clients’ mental health, especially when clinicians and clients engaged in collaborative data interpretation. However, clinicians experienced several mismatches between system usability and their technical competencies, and many clients found it difficult to comply with the self-assessments. Importantly, most participants wanted to use digital self-monitoring tools in the future.

**Conclusions:**

Clinicians’ and clients’ choice to adopt and integrate self-monitoring tools in their practice seems to depend upon the perceived balance between the added benefits and the effort required to achieve them. Enhancing user support or redesigning ESM tools to reduce workload and data burden could help overcome implementation barriers. Future research should involve end users in the development of ESM self-monitoring tools for mental health care and further investigate the perspectives of nonadopters.

## Introduction

To improve access to and facilitate person-centered mental health care, digital technologies are increasingly being deployed to collect and share health-related data, deliver care, and support individuals in managing their health [1,2]. The experience sampling method (ESM; also termed ecological momentary assessment) is a structured diary technique that enables individuals to collect detailed information about their mental health in their daily lives [3]. Using smartphone apps, the ESM prompts individuals to complete brief self-assessments multiple times daily, assessing their emotional states, behaviors, and psychological symptoms. By allowing individuals to collect this information and share it with their clinicians, the ESM can ensure that therapeutic decisions are aligned with clients’ everyday experiences and needs. For example, by providing insights into which activities an individual commonly engages in, and how they respond to different daily life situations and stressors, clients and clinicians can make shared decisions about the focus of treatment [4].

Despite its potential, the ESM, similar to many other digital mental health interventions [5,6], struggles to move from piloting and trialing to actual clinical implementation. To address this research-to-practice gap, scholars have made calls to adopt user-centered design strategies for developing digital mental health tools [7]. User-centered design emphasizes the importance of conducting in-depth analyses of users’ goals, needs, and context of use to inform technology design [8]. Nonetheless, most evaluations of digital health technologies focus primarily on quantifiable, technical aspects such as system performance, usability scores, and cost benefits [9,10]. However, an often overlooked but crucial step in implementing the ESM and self-monitoring tools is understanding how and why people integrate these technologies into their daily practices [11]. For example, what are the goals people hope technologies will help them achieve, what difficulties do individuals encounter when using new technologies, and what strategies do they apply to overcome technology use obstacles?

Studying the situated use of the ESM can provide essential knowledge into how users appropriate and make sense of these tools [12,13]. Technology appropriation can be examined in three main stages [13]. The first stage involves users’ prior knowledge and expectations, which determine their initial willingness to adopt the technology. Expectations about potential benefits (eg, simplifying tasks) and the effort required (eg, time spent on training) are particularly crucial in shaping the initial interest [14,15]. In the second phase, users explore and evaluate the technology’s capabilities, while becoming familiar with its functions. They adapt their practices to integrate the technology (eg, maintaining an internet connection), and adapt the technology to suit their needs (eg, disabling certain features or altering settings) [13]. They will test the technology’s applicability in different situations and determine what features are most useful to them. Finally, if users find that the technology has added value and helps them achieve their goals and tasks, they might develop new routines and workflows that allow them to integrate and use the technology in their daily practice, thus moving into the third stage of persistent use [13].

To date, the appropriation of ESM tools in health care has largely been overlooked. While one study has examined how individuals with back pain appropriated ESM tools for pain management [16], there is a lack of research in the field of mental health care. As a consequence, we have a limited understanding of how individuals with mental illnesses and their clinicians interact with and make sense of digital ESM tools, and what might lead them to adopt or abandon these technologies. However, understanding this is vital for facilitating successful implementation [15,17]. To address this knowledge gap, we undertook a pilot implementation study, using the ESM tool “IMPROVE,” a clinical prototype tool informed by research examining clinicians’ and clients’ design preferences [18,19]. IMPROVE was used as a part of therapy following a 3-step intervention, in which clinicians and their clients could (1) personalize the tool to clients’ specific problems and situations, (2) self-monitor clients’ mental health and daily activities via an app, and (3) review the collected data in summarized graphs in an online dashboard. To inform the further development and implementation of clinical ESM tools, this paper aimed to evaluate participants’ (1) prior knowledge and expectations, (2) actual use in practice, and (3) potential future integration of ESM-based self-monitoring tools. Addressing these gaps in the literature will provide valuable insights into why and how clients and clinicians in mental health care choose to adopt or abandon digital self-monitoring tools in therapy.

## Methods

### Ethical Considerations

All participants provided written informed consent, and all procedures were approved by the medical ethics committee of KU Leuven (S64244). Participants were given a study ID to ensure anonymity, and interviews were pseudonymized during the transcription by removing personally identifiable information and replacing them with pseudo codes. Finally, participants were allowed to freely use the IMPROVE tool after completion of the study but were not given additional compensation.

### Study Design and Recruitment

We conducted a pilot study within the University Psychiatric Center KU Leuven in Belgium in which we involved clients and clinicians in testing and evaluating IMPROVE. The research team was unable to access the clinic directly, due to COVID-19 restrictions. Therefore, invitations to participate were emailed to all psychiatrists and psychologists affiliated with the psychiatric center (N=142). The study aimed to enroll 12 clinicians, and initial invitations were followed by up to 2 reminder emails. For those interested, online informational sessions with the research team were organized. Participating clinicians were asked to recruit at least 1 client from their practice. By allowing clinicians to select clients, the referred sample provided a practical representation of the clients with whom clinicians were likely to use the tool within a real-life context.

The inclusion criteria were kept broad to reflect the diverse reality of clinical practice. To be eligible, clinicians needed to be certified mental health professionals and proficient in Dutch, which was the language the intervention was designed in. Clients had to be 18 years or older and also proficient in Dutch. To replicate real-world implementation, clients were asked to use their own smartphones. As a result, only clients owning a smartphone with at least 3G coverage were eligible to participate.

**Figure 1.**
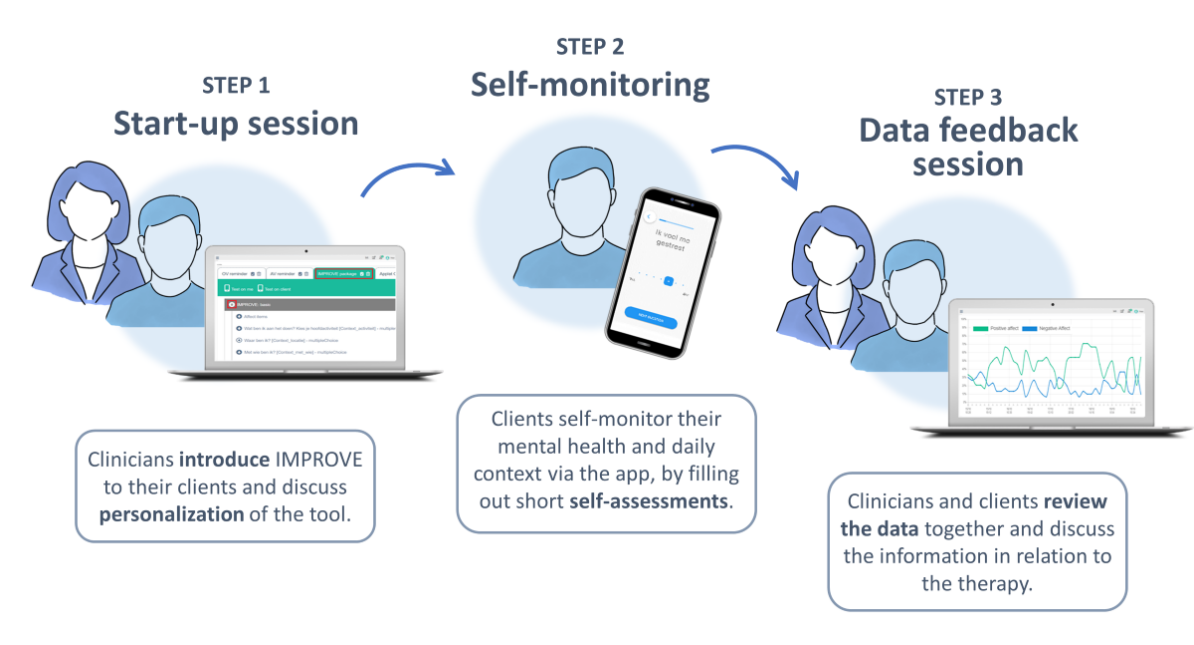
Overview of the steps included in the IMPROVE intervention.

IMPROVE was run on the digital platform m-Path [20]. To enable clinicians to use IMPROVE, they received a manual with instructions on using the tool in their practice (Multimedia Appendix 1: IMPROVE training manual) and were invited to join an online training session. Clinicians and their clients were then requested to complete the 3-step intervention (Figure 1). In the first step, clinicians introduced IMPROVE to their clients and assisted them in downloading the app on their smartphones. They also discussed personalization of the tool, such as adding symptom-specific questions or adjusting the notification schedule. In the second step, clients self-monitored their mental health for 6 consecutive days. They received 10 semirandom notifications daily, prompting them to complete brief self-assessments on mood and context. Additionally, they received a morning notification to assess their sleep and an evening notification to evaluate the day. In the third step, clinicians and clients reviewed the collected data together, exploring factors such as clients’ activities, mood variability, and mood-context interactions.

### Data Collection and Analysis

Due to COVID-19 measures, data were collected remotely via online surveys in RedCap (Research Electronic Data Capture, Vanderbilt University) [21] and through Skype interviews (Version MSO; Skype Technologies). In some cases, poor connections affected the quality of the interview recordings; however, the overall data collection was not hindered by remote methods. Demographic information was collected at study enrollment and participants who completed the intervention were invited to participate in a semistructured interview (Multimedia Appendix 2: Interview guides). Interviews were audio recorded and transcribed verbatim. We performed a thematic analysis [22] in which we first conducted top-down content-coding of the transcripts using a thematic framework that identified interview material related to clinicians’ and clients’: (1) previous knowledge and expectations, (2) actual use in practice, and (3) potential future integration of the IMPROVE tool (Table 1). Hereafter, we performed additional inductive in vivo coding and used pattern and focused coding to create labels for the in vivo codes and compiled them into additional subthemes [23]. LdT undertook all primary and secondary coding, which was then revised by the coauthors and discussed in multiple peer debriefing sessions [24]. After each session, the coding categorizations and labels were modified and refined based on feedback from coauthors. To explore the potential impact of the different themes on the discontinued use of IMPROVE, additional information was extracted from informal participant contact records kept by the research team.

**Table 1.** Thematic framework for top-down coding of interviews.

Theme	Description
Prior knowledge and expectations	Participants talk about any prior experiences or knowledge they have regarding the ESM^a^ or self-monitoring tools (analog or digital). Participants talk about their motivations and expectations related to using the ESM or self-monitoring tools and participating in the study.
Actual use in practice	Participants talk about how they used the IMPROVE tool and how they judged these experiences. Mentioning eg, what purpose they used it for, when, and how often they used it.
Potential future integration	Participants talk about their interest in using the IMPROVE tool (or similar tools) in the future. Participants talk about potential changes that they find would be relevant for future use and integration of the IMPROVE tool.

a ESM: experience sampling method.

## Results

### Participant Characteristics

Nineteen clinicians expressed interest in participating in the study, of whom 12 were enrolled. While a systematic record of the number of clients approached by clinicians was not maintained, at least 29 clients were invited, with 24 agreeing to participate. Of the enrolled participants, 8/12 clinicians (67%) and 17/24 clients (71%) completed the intervention; among these, 7/8 clinicians (88%) and 11/17 clients (65%) subsequently participated in an interview. A demographic summary of interview participants can be consulted in Multimedia Appendix 3: Demographic summary.

### Thematic Analysis

A complete overview of the themes and subthemes of the analysis and how frequently they were mentioned by participants can be found in Multimedia Appendix 4: Overview of themes. Tabulated in vivo code summaries of participants’ individual experiences can be found in Multimedia Appendix 5: Individually summarized experiences. Additionally, illustrative summaries are displayed in text boxes.

### Previous Experience and Expectations

Individuals’ experiences and expectations are important indicators of their initial willingness to adopt technologies. While only 1 clinician had experience using digital self-monitoring tools, most clinicians and some clients had experience using analog self-monitoring or diary techniques. Some clinicians also had experience with different forms of digital tools within their practice (eg, online training platforms, video consultations, and virtual reality). Similarly, several clients had experience using mental health apps, eg for practicing breathing techniques and cognitive-behavioral therapy exercises. Despite their limited experience, clinicians generally expected that IMPROVE would offer benefits over analog registration methods, allowing them to more easily collect and summarize information about their clients. However, several clinicians also anticipated that there would be challenges and limitations for using the tool. The most commonly mentioned was that low digital literacy could be a barrier for both clients and clinicians (Clinician 0100 Textbox 1). Clinicians’ motivation for testing the tool was primarily driven by curiosity and the conviction that they need to master digital tools, as these are becoming increasingly dominant in the health care sector. Interestingly, most clients were primarily motivated by a wish to help and contribute to research but also expressed a curiosity toward what digital mental health tools might offer (Client 0501 Textbox 2). Some clients hoped that IMPROVE would allow them and their clinicians to get a better understanding of their mental health problems.

Textbox 1. **“Might use digital self-monitoring tools in the future”– selected participants’ experiences.**

CLINICIAN 0100:

Prior knowledge and expectations

- I had no experience with digital self-monitoring, but I am currently testing VR technology in therapy. I have previously worked with analog self-registration in therapy.
- I expected it would be technically challenging to work with the tool.
- I want to try using new methods in therapy and I was eager to try the tool.

Actual use in practice

- It was difficult to find things in the dashboard and I needed help from the research team to use the tool. After a while using the platform got easier, but it required a lot of time to get started.
- It was difficult to draw conclusions about my clients' data, but I asked my client for clarifications when I was not able to interpret their data.

Potential future integration

- The tool gave me more information about what happens in clients' lives.
- I might use the tool again if it is made easier to use.

CLIENT 0503:

Prior knowledge and expectations

- I had no experience with self-monitoring apps.
- I hoped to gain more insight into my mental health.

Actual use in practice

- Assessment frequency was high, but okay for 1 week.
- I think I responded to almost all notifications, but I sometimes missed notifications because I forgot my phone. Also, when I was not feeling well I didn't respond to the notifications.
- Identifying, labeling, and scoring emotions on a scale are difficult.
- My therapist had difficulties operating the dashboard, so we didn't go into detail with the data.

Potential future integration

- I might use the tool again, but it is tiring to do for a long time.
- I prefer using the tool as a part of therapy, but I would like to have access to the data myself.

CLIENT 0802:

Prior knowledge and expectations

- I have no experience with health apps, but I use analog methods to keep track of my mental health.
- Digital tools allow you to do more than analog tools, so I thought it would be interesting to try.

Actual use in practice

- The number of notifications was okay, but I sometimes missed notifications because I was working or sleeping.
- Using the tool during the therapy session made it easier to recollect things that had happened.

Potential future integration

- I might consider using the tool again.

Textbox 2. **“Not likely to use digital self-monitoring tools in the future”– selected participants’ experiences.**

CLIENT 0501:

Prior knowledge and expectations

- I had no experience with self-monitoring apps.
- I was hesitant to participate because I do not like self-assessments.
- I thought it was worth trying and I wanted to contribute to improving mental health care.

Actual use in practice

- It was difficult always to remember to have my phone with me.
- Notifications were sometimes disturbing and the assessment frequency was too high.
- It was important to me that I did not miss notifications; therefore, fear of missing notifications would sometimes stress me.
- Self-reflection was sometimes difficult and confronting.
- It was interesting to look at my data.

Potential future integration

- I don't find it likely that I will use the tool again.

### Actual Use in Practice

Participants’ willingness to test the IMPROVE tool was influenced by their initial expectations that digital self-monitoring would provide them with benefits and advantages. Below we describe how clinicians and clients used the tool in practice and how they evaluated its capabilities and contextual fit.

#### Step 1: Start-Up Session

Clinicians generally found that the time investment required to learn to use IMPROVE was too much considering the time they had available. Experiencing time constraints was also one of the most common reasons reported by clinicians who did not complete the intervention. Personalizing clients’ questionnaires in particular asked for extra time and effort from clinicians. Despite emphasizing that the personalization of the IMPROVE questionnaires made the tool more relevant, clinicians made limited use of the available personalization options. Most clients also expressed that personalization of the tool is desirable but that clinicians did not discuss this with them.

Furthermore, while most clinicians were convinced that using the IMPROVE tool would become easier with practice, many clinicians indicated that the complexity of the tool was too high. In particular, clinicians found that navigating the dashboard was not intuitive and that it was difficult to find things and set up the clients’ questionnaires. Thus, many experienced that they did not have adequate competencies to make full use of the tool; “I constantly felt I was doing something wrong,” 1 clinician explained. However, at the same time, clinicians made limited use of the training manual and expressed a wish for more in-person support. This was also reflected by several clinicians contacting the research team for additional support after the initial training (Clinician 0100*,* Textbox 1). Interestingly, some clinicians resolved their need for support by organizing training sessions with colleagues.

#### Step 2: Self-Monitoring

The majority of clients reported that they made an effort to comply with the notifications, but many found that the frequency of the self-assessments (10 notifications per day) was too high. A few clients indicated that they would consciously skip assessments because they did not consider it important to respond to all notifications. Conversely, some clients also mentioned that missing notifications would make them feel guilty and annoyed, or that the thought of missing assessments made them nervous and stressed (Client 0501, Textbox 2). Although clients reported that completing the assessments did not take them long, many experienced difficulties responding to the notifications within the fixed 15-minute response window. Several reasons for this were voiced, of which being occupied with work and other daily activities was the most frequent (Client 0802, Textbox 1; Client 0601, Textbox 3). Having an irregular day rhythm or sleep schedule that did not match the notification schedule, needing to carry one’s phone and ensure an internet connection, and feeling unwell or tired were also factors that made complying difficult. Importantly, clients who dropped out of the study also reported a lack of time, difficulties complying with the assessments, notifications stress, and feeling unwell as reasons for not completing the intervention.

Textbox 3. **“Likely to use digital self-monitoring tools in the future”– selected participants’ experiences.**

CLINICIAN 0600:

Prior knowledge and expectations

- I have no experience with digital self-monitoring, but I have used analog self-monitoring techniques in therapy.
- I think digital tools are easier to use than analog self-monitoring methods. Digital tools are used more and more, and we need to offer clients tools that can help them achieve their goals.

Actual use in practice

- It takes time and effort to learn to use the tool.
- I didn't personalize my client questionnaire, because I wanted to start with the basics.
- I monitored my clients' responses and contacted them if I could see they weren't responding.
- There was a lot of data; I started with simpler visualizations and gradually added things. If I didn't know the client beforehand, I would not be able to make sense of the data.

Potential future integration

- The tool provided more details and an overview and allowed us to go more in-depth with problems that we already knew were there. We used the tool to identify what was important for the client.
- I will certainly consider using this tool again with my clients.

CLIENT 0601:

Prior knowledge and expectations

- I had no experience with health apps.
- I had no specific expectations and I’m generally skeptical about health apps, but I wanted to help research.

Actual use in practice

- I responded to as many notifications as I could, but I missed a lot because I was busy. I didn't consider it a problem that I missed some notifications. I also had to remember to bring my phone and connect it to 4G.
- It is sometimes difficult to assess whether your mood changed and how much.
- I was amazed to see what came out of the data. My therapist was able to do a lot with the data, despite my low compliance.

Potential future integration

- The tool provided useful information and allowed me to get to know myself better.
- I would be interested in using the tool again, but I would like to have access to the data myself.

CLINICIAN 1200:

Prior knowledge and expectations

- I used digital self-monitoring tools before in therapy.
- Digital self-monitoring tools are easier to use and give better insight into clients’ lives than other methods. However, self-monitoring can be difficult for some clients.

Actual use in practice

- Setting up the tool took a lot of time.
- It was interesting to look at the data, but the many visualizations were somewhat overwhelming.

Potential future integration

- The tool quickly provides you with an overview of how your client is doing.
- I would like to use the tool again, but it should be made less burdensome for clients. It would be interesting to monitor clients for a longer period to evaluate their progress and the effect of treatments.

CLIENT 1201:

Prior knowledge and expectations

- I had no experience with mental health apps.
- I expected the tool would make it easier for my therapist to understand my problems.
- I think there is a need for more mHealth in mental health care and that this tool might help me and others.

Actual use in practice

- There are too many notifications and they are sometimes disturbing. People should be allowed to snooze notifications if they are busy.
- I did my best to respond to the notifications. On good days, it is okay to complete the assessments, but on bad days, it is difficult.
- My therapist identified moments when I was feeling bad and tried to understand these.

Potential future integration

- The tool can make you aware of what you need to work on, and it makes it easier to monitor the effects of your treatment.
- I would like to keep using the tool to check how I am doing.

Several clients found the practice of labeling and rating their emotions difficult. Some indicated that they were unsure whether they completed the self-assessments correctly and expressed a need for more guidance from their clinicians on how to respond to the assessments. Several clients also expressed a wish for more open questions that would allow them to describe their experiences in more detail, as they did not find that the default questions were sufficiently able to capture their experiences. Interestingly, while clinicians were not instructed to do so, some monitored their clients’ responses during the self-monitoring week as a form of remote monitoring of clients (Clinician 0600, Textbox 3).

#### Step 3: Data Feedback Session

To get the most out of the data feedback session, most clinicians reviewed their clients’ data in preparation for the session. Many felt that preparation was necessary for them to understand the data and be able to discuss it with clients. Many clinicians found it challenging to interpret and navigate the graphs, which they often perceived as overwhelming. This generally led clinicians to be selective in the data they would review and discuss with clients. All clinicians reviewed the data with their clients and some also encouraged their clients to give their interpretation of the data or to provide further clarification (Clinician 0100, Textbox 1*)*. Similarly, clients indicated that the support of a clinician was crucial for them to make sense of their data. While most clients reported that they could understand the graphs, they also indicated relying on their clinician to help them interpret the data. These findings indicate that, despite challenges, most clinicians could provide useful feedback to their clients based on the self-monitoring data.

### Potential Future Integration

Despite encountering challenges, both clinicians and clients described that the IMPROVE tool provided them with useful insights. Next, we will explore how these experiences influenced participants’ willingness to integrate and use digital self-monitoring tools in their future practice. Clinicians and clients were generally open to using digital self-monitoring tools again. Participants who expressed the greatest interest in continued use more often experienced an added value of using IMPROVE (see Textbox 3). This included having access to more detailed information about clients’ mental health, which helped create an overview and clarity, and identify focus points to discuss in therapy. Clinicians who had initial positive expectations and believed digital tools were important in supporting mental health care were also more willing to use digital self-monitoring again. The clinicians who were more hesitant reported more difficulties using IMPROVE and expressed a greater need for support (see Textbox 1).

Clients who expressed a low interest in using digital self-monitoring tools in the future also reported negative reactivity in the form of stress and increased negative emotions during self-monitoring (see Textbox 2). Interestingly, all these clients had high compliance during the self-monitoring. However, clients overall reported that the assessment frequency should be lowered to make future self-monitoring less burdensome. Moreover, some clients and clinicians expressed an interest in using the tool for extended periods to monitor progress and the effects of therapy. Finally, clients and clinicians also expressed a desire to adapt the self-assessment further and monitor factors more specific to the individual client.

## Discussion

### Principal Findings and Implications

To guide the successful implementation of digital self-monitoring tools in mental health care, it is pivotal to understand how people react, respond, and adapt to these technologies. We examined how clinicians and clients in a psychiatric center appropriated the ESM-based self-monitoring tool IMPROVE within their therapeutic practices. In line with existing theories, our results indicate that clinicians’ and clients’ willingness to adopt and integrate digital self-monitoring tools into their practice depend on the perceived balance between added benefits and the effort necessary to accomplish these [17]. The main benefits identified in our study included better information about clients’ mental health, while system usability and assessment burden demanded extra effort from participants. Below we discuss the potential implications of this for the implementation of the ESM tools in clinical practice.

While some struggled more than others, all interviewed participants managed to reach a basic level of use of the IMPROVE tool within the intervention period. Furthermore, we observed a great interest and curiosity towards digital mental health tools, especially among clinicians. Many clinicians experienced that using IMPROVE added value and generated more clarity and focus in the therapy, by providing more detailed information about the clients’ mental health. Several clients also experienced these benefits. Our findings suggest that a collaborative effort between clients and clinicians is key to maximizing the experienced benefits of the ESM tools. While many clinicians struggled to construct a frame for the interpretation of clients’ data, several turned to their clients to help make sense of the data. Clients also stressed the importance of having a clinician support them in interpreting their data and doubted whether they would be able to do this themselves. Other studies similarly highlighted the value of patients providing clinicians with additional information about patient-generated health data to unveil the subjective meanings of the data [25]. This indicates that collaborative data interpretation is an essential component in the clinical application of the ESM tools, which boosts the perceived usefulness of the tool. Therefore this component should be emphasized and strengthened in future implementation initiatives.

Most participants in our study expressed willingness to use the ESM or similar tools in the future, although many emphasized the necessity of making changes and improvements to the tool. This aligns with previous findings that clinicians and clients are generally interested in the ESM-based self-monitoring [18,19,26]. However, the need for further adaption was reflected by participants encountering several challenges in using and integrating IMPROVE into their practice. Many clinicians experienced difficulties navigating the tool’s functionalities, which made it difficult for them to start using the tool and apply more advanced features, such as personalizing clients’ questionnaires. Clinicians, therefore, expressed a need for more in-person support. Unfortunately, these types of usability issues are common in digital health tools [27]. A study examining usability problems of eHealth applications found that system navigation, interface design, and lack of built-in guidance and support accounted for 69% of users’ usability issues [27]. Furthermore, consistent with other research [28,29], our study indicated that technology literacy and attitude can influence willingness to use digital tools. More anthropological field research on mental health care workers’ existing technology habits and literacy might help inform the system design of the ESM tools and understand users’ context and capabilities. Similarly, engaging end-users in co-design processes could help tackle usability issues that could lead users to abandon the tools. Finally, our results show that adequate support for clinicians and clients is needed to facilitate the implementation of the ESM tools in mental health care. Especially in the early adoption phases, users should be supported in familiarizing themselves with the tools and integrating them into their work routines. Potential solutions could include more built-in guidance functions in the tool or establishing additional structures (eg, service centers) that can provide direct user support.

Furthermore, our findings emphasize that self-monitoring demands a lot of clients, and can be difficult and burdensome for people with mental health problems. While clients expressed a wish to comply with the self-assessments, they often found it practically challenging. In line with other self-monitoring studies [30], we found that competing activities (eg, working) and technical issues (eg, device or internet access) were the most common reasons for missing assessments. To comply with the assessments, several clients had to change their phone habits, eg, ensuring that they always had their phone with them, and had notifications and internet connection on. As it is known that existing habits are important predictors of technology acceptance [17], this need to change habits can become a threat for sustained clinical implementation of ESM tools. Furthermore, some clients reported negative reactivity to self-monitoring, which was associated with less interest in using the tool again. Other studies also found that while self-monitoring can be motivating and helpful, it can also become a stressful activity that clients feel obliged to comply with [31]. This highlights the need to investigate how self-monitoring tools can be made less burdensome for users while still producing valuable information. Potential solutions could include the integration of passive monitoring [32,33] or adaptive assessment schemes that allow for periods with lower and higher assessment intensity [34,35].

### Limitations

The findings of the study should be interpreted considering the following limitations. One limitation is that the study relied on referral sampling, which might have influenced the representativeness and diversity of the sample. Furthermore, the study was conducted in a large psychiatric center of a university in Belgium. Clients and clinicians within different health care settings or with different demographic backgrounds might have different experiences of using digital self-monitoring tools. Another limitation is the potential overrepresentation of people with a positive attitude towards digital mental health tools. Several clinicians for example recruited clients who they expected were interested in using digital tools and had the necessary technical literacy. Furthermore, none of the participants who dropped out of the intervention participated in the interviews. However, using contact records data, we established that some of the challenges experienced by participants who were interviewed were also reported as reasons for dropout by participants who did not complete the intervention. Future research should aim to better understand the views of nonadoptors, identify the main reasons for nonadoption, and work towards tackling these barriers in the design and implementation of ESM self-monitoring tools.

### Conclusion

ESM-based self-monitoring can provide clinicians and clients with useful information about how clients’ daily life activities correspond to fluctuations in their mental health. These benefits seem to increase when clinicians and clients engage in collaborative interpretation and sense-making of the clients’ data. Therefore, ESM tools have a clear potential to support a person-centered approach to mental health care rooted in clients’ daily life experiences. However, our study highlights that the effort required by clinicians and clients to integrate ESM tools into daily practice remains substantial due to system usability challenges and the burden of repeated assessments. Addressing these issues in future ESM tool developments will be crucial for successful implementation. One potential solution to tackle these barriers is enhancing user support. Another is modifying key features of ESM tools to better suit users and their contexts, such as reducing the frequency of assessment and data information load. Engaging end-users directly in this process through user-centered design approaches may ensure a better fit between the tools, their context of use, and user goals and ultimately facilitate better adoption.

## Supplementary material

10.2196/60096 Multimedia Appendix 1 IMPROVE training manual.

10.2196/60096 Multimedia Appendix 2 Interview guides.

10.2196/60096 Multimedia Appendix 3 Demographic summary.

10.2196/60096 Multimedia Appendix 4 Overview of themes.

10.2196/60096 Multimedia Appendix 5 Individually summarized experiences.

## Abbreviations

ESM: experience sampling method
