# Supplementary figures and images for: Understanding Appropriation of Digital Self-Monitoring Tools in Mental Health Care: Qualitative Analysis
Source: JMIR Hum Factors. 2025 Mar 3;12:e60096. doi: 10.2196/60096 (PMC11892539; doi:10.2196/60096)

**Multimedia Appendix 1 - IMPROVE training manual**
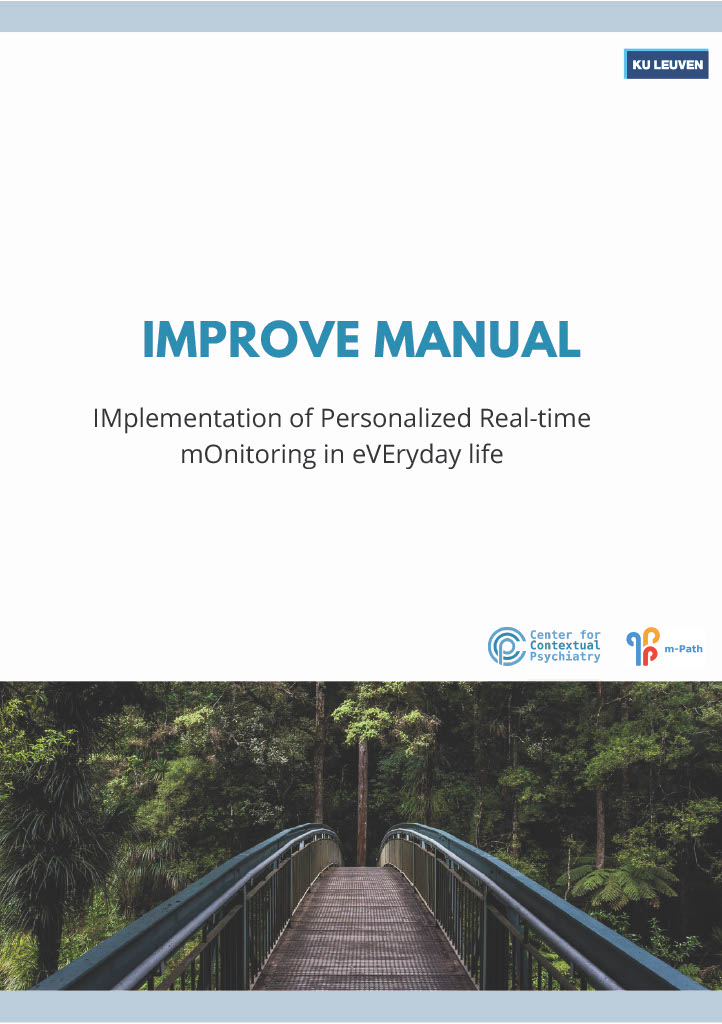


**
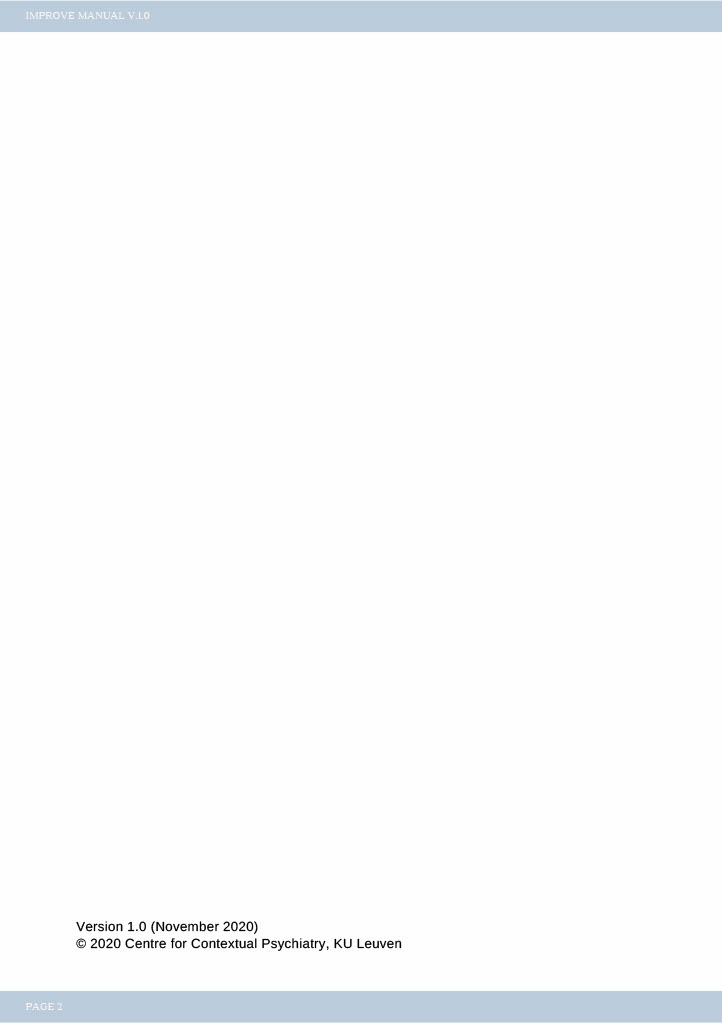

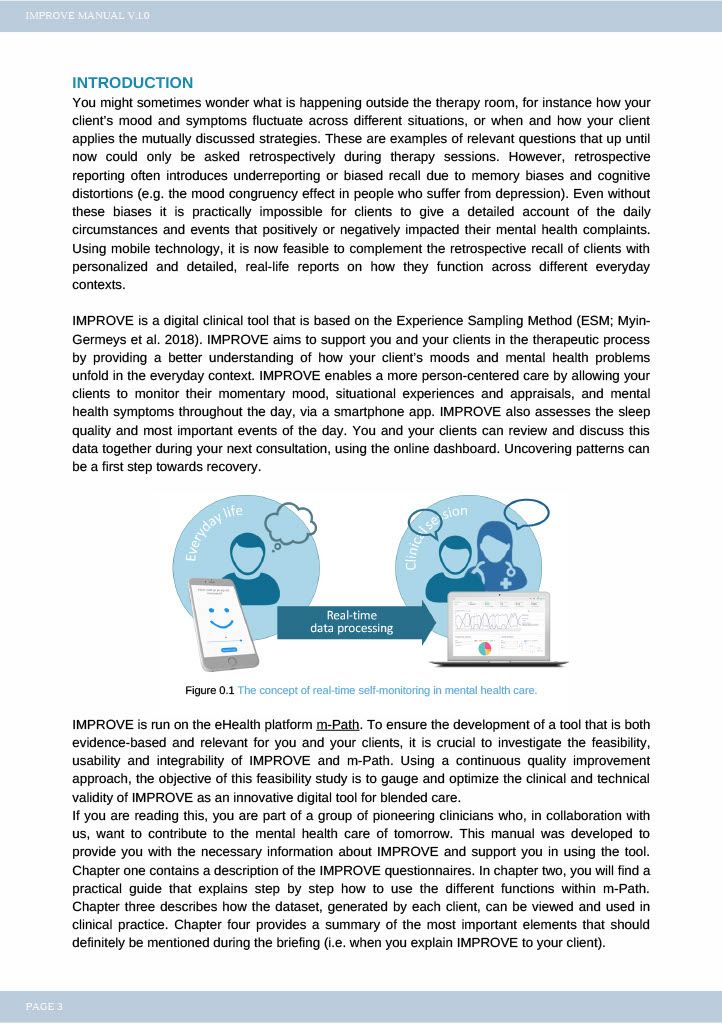

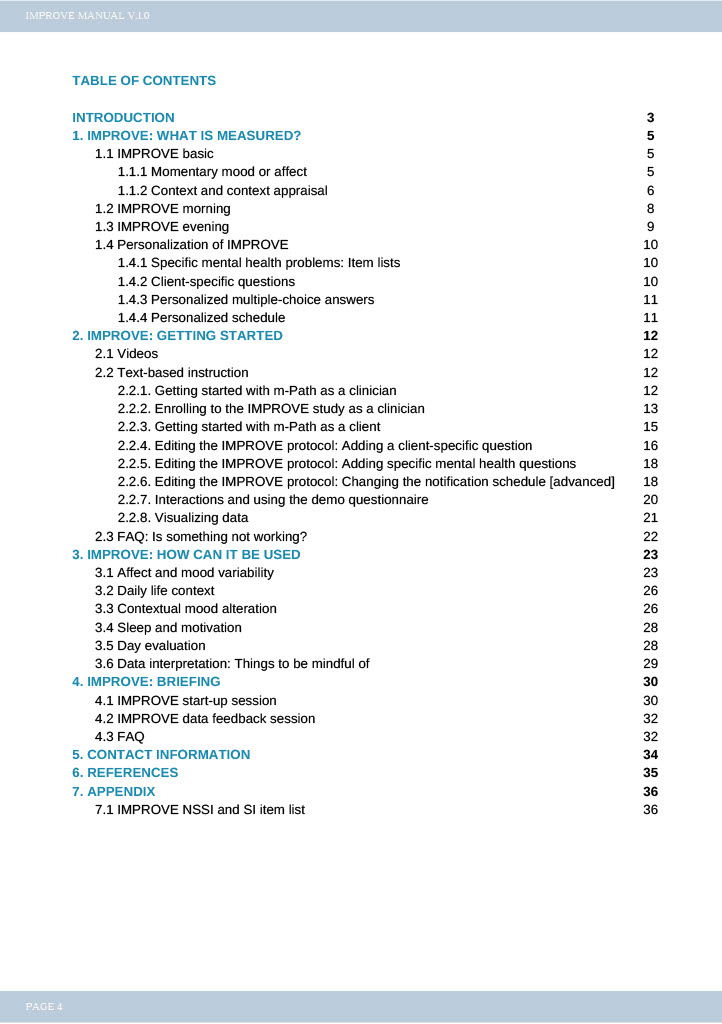

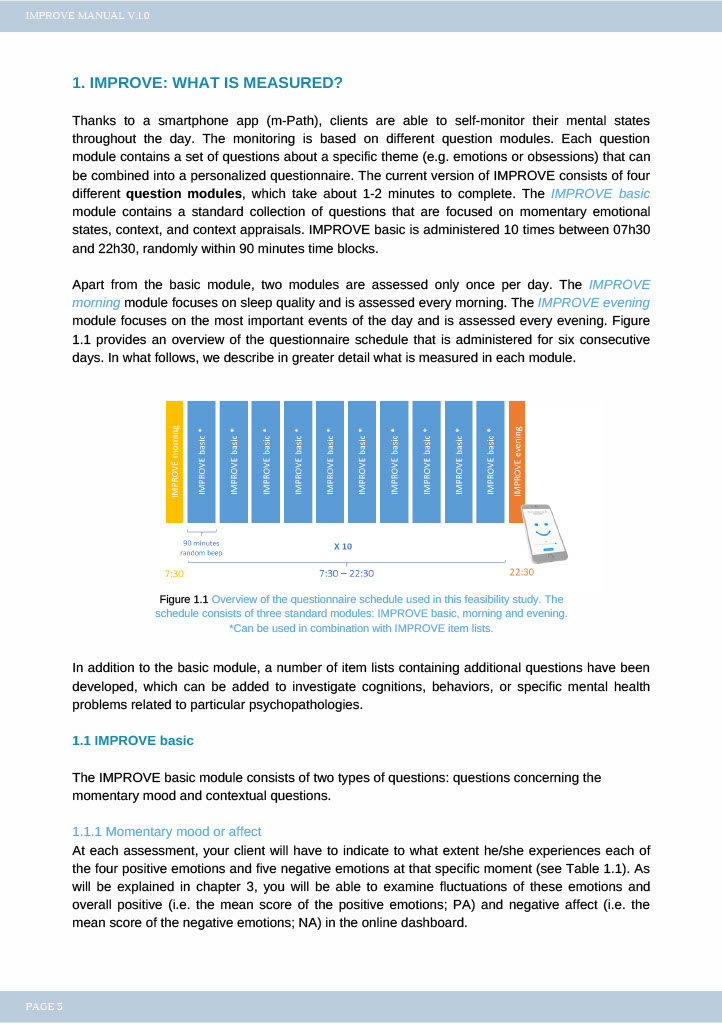

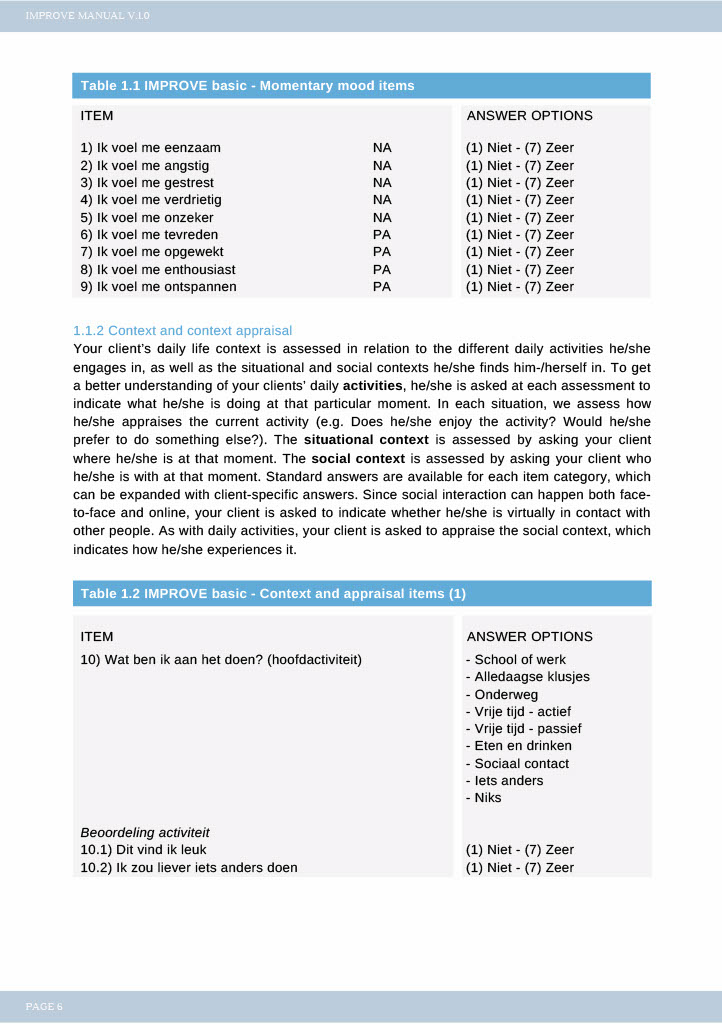

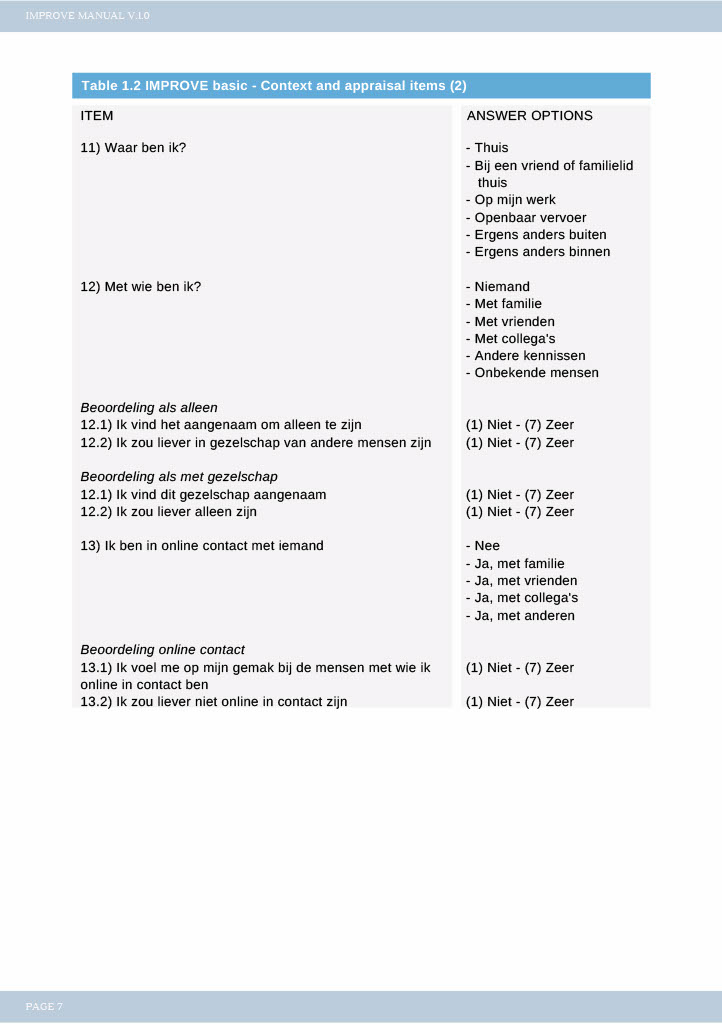

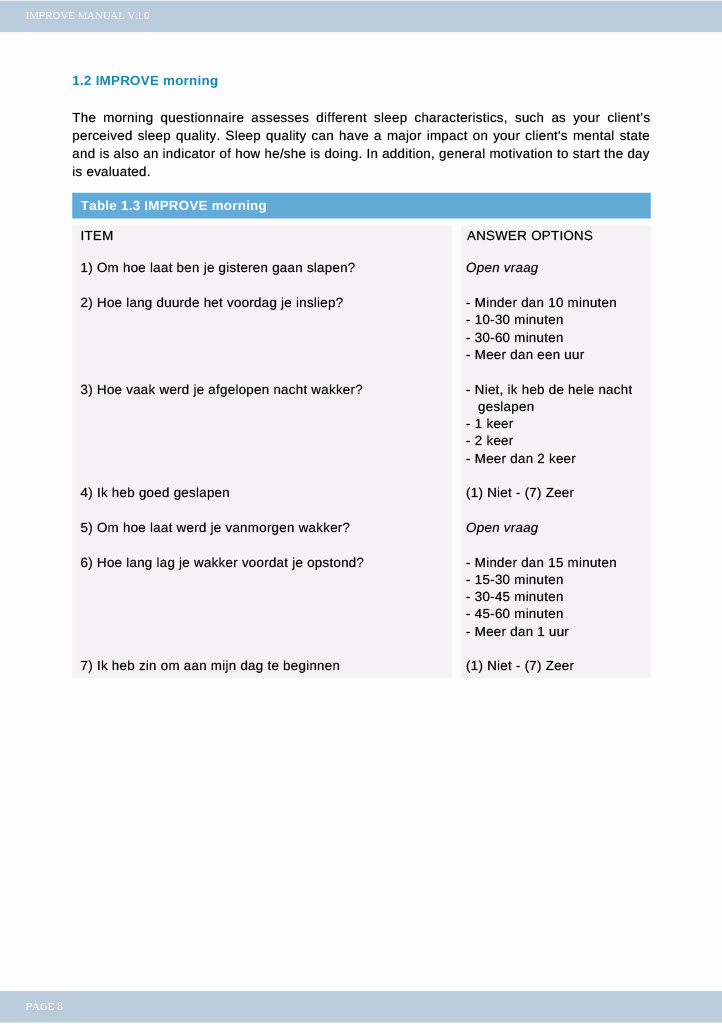

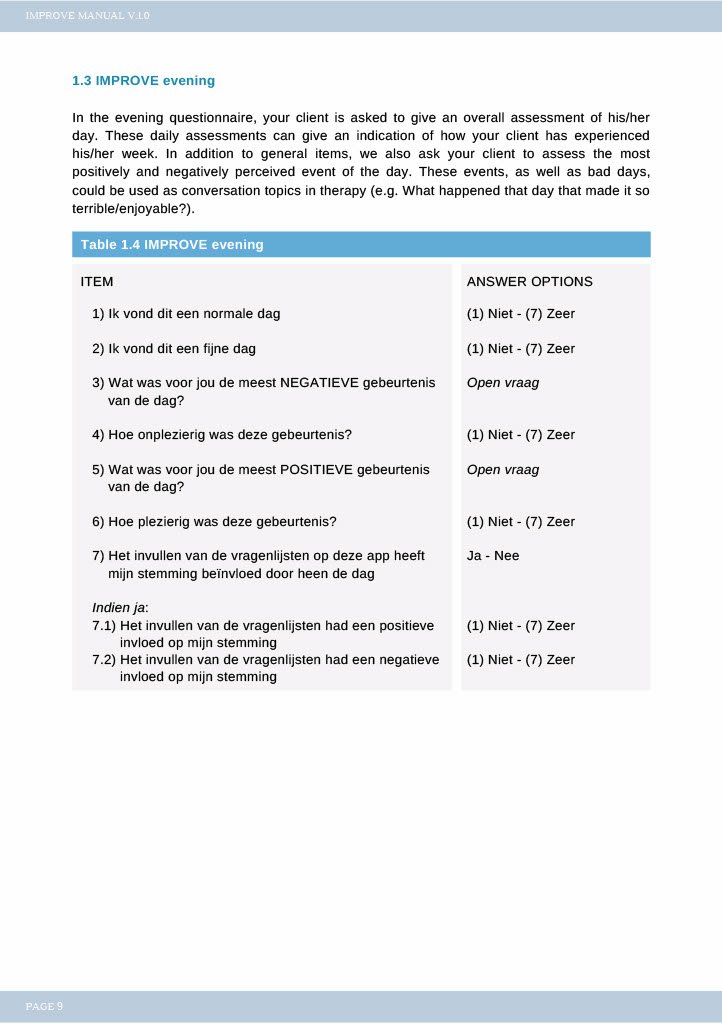

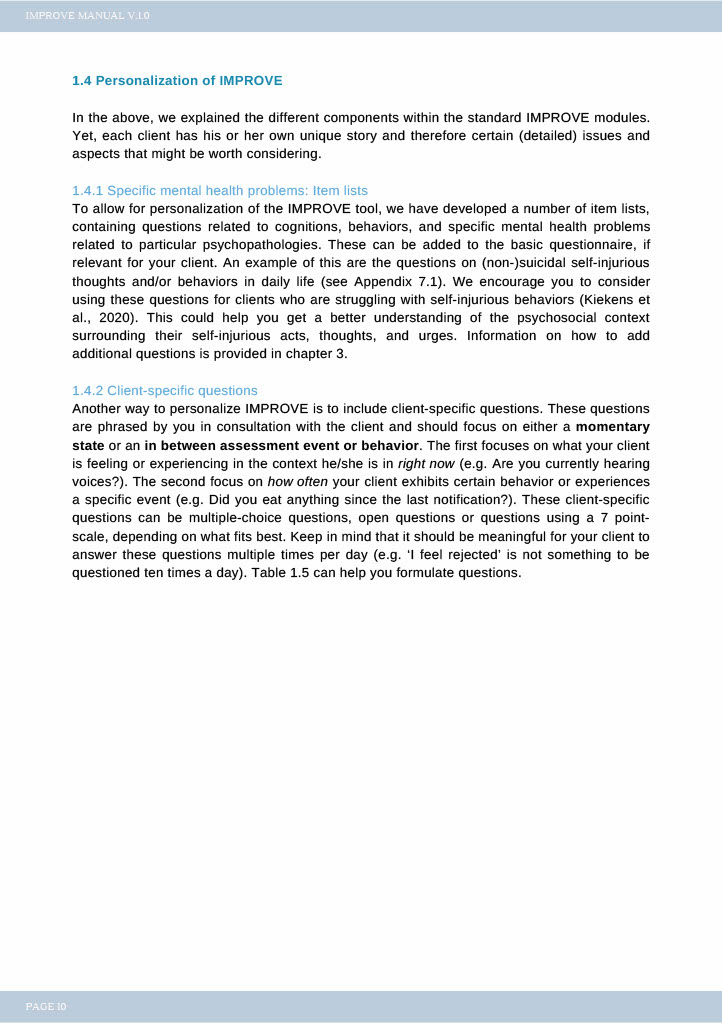

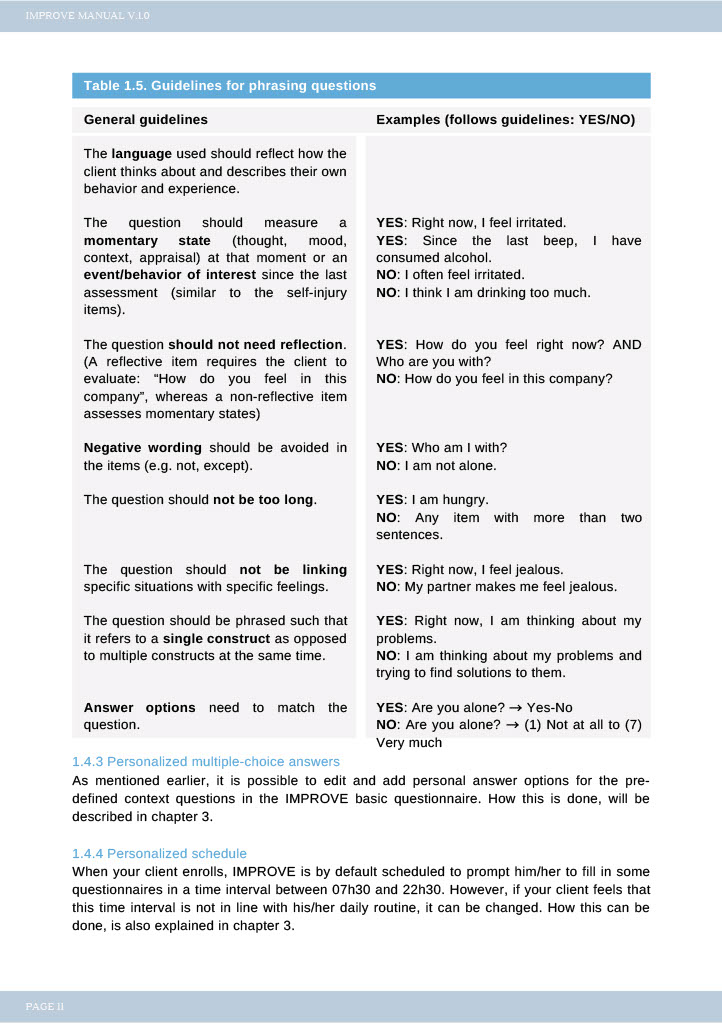

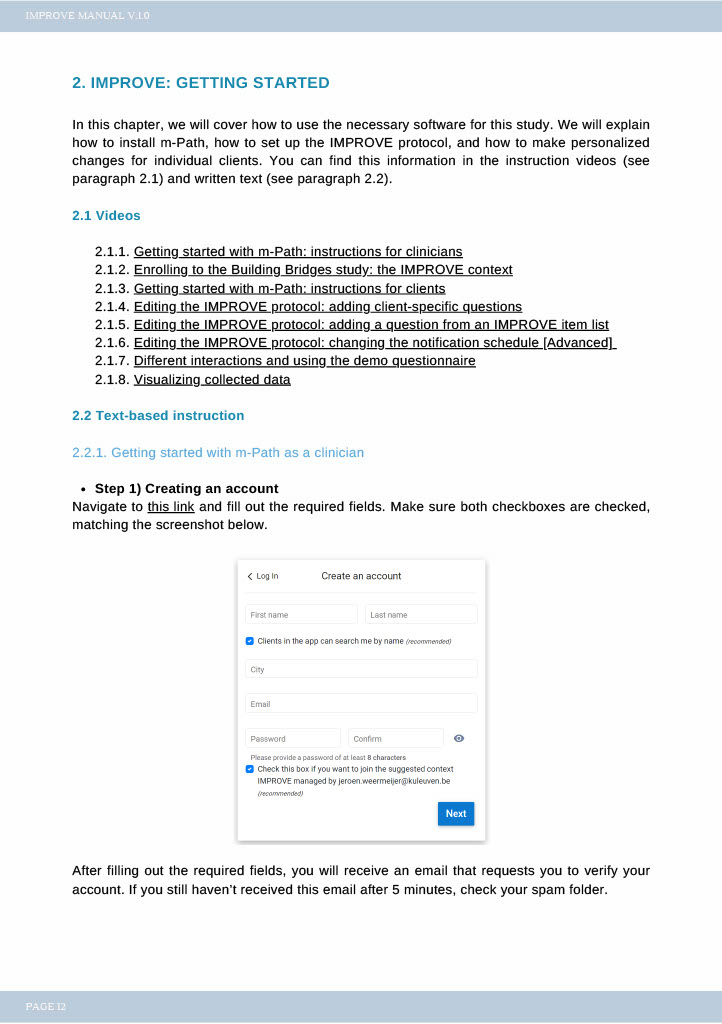

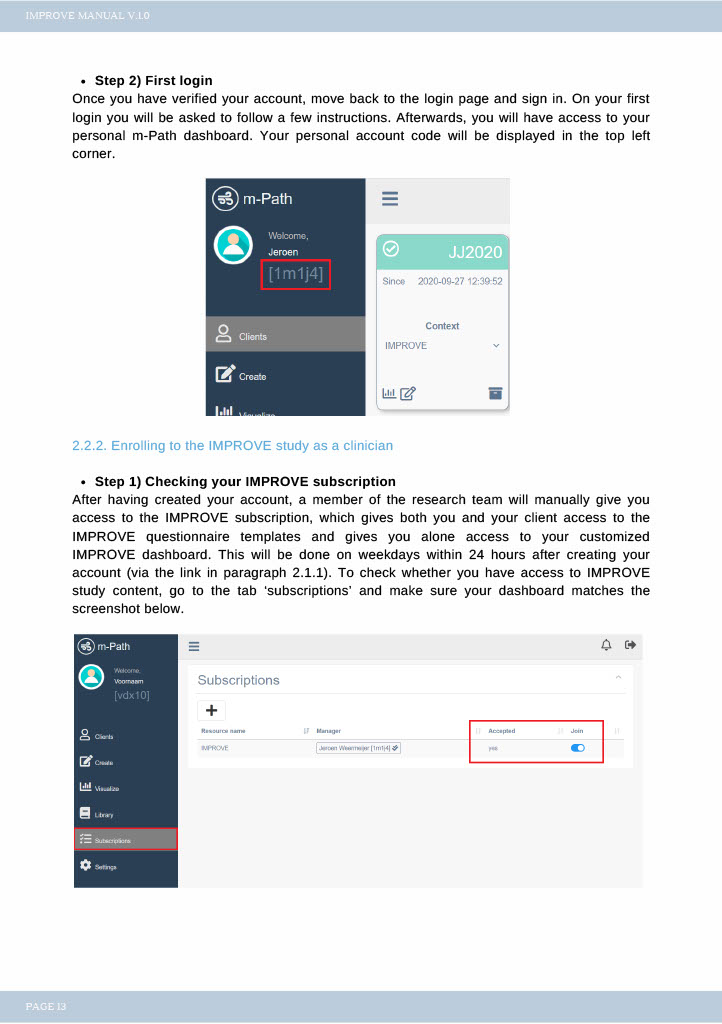

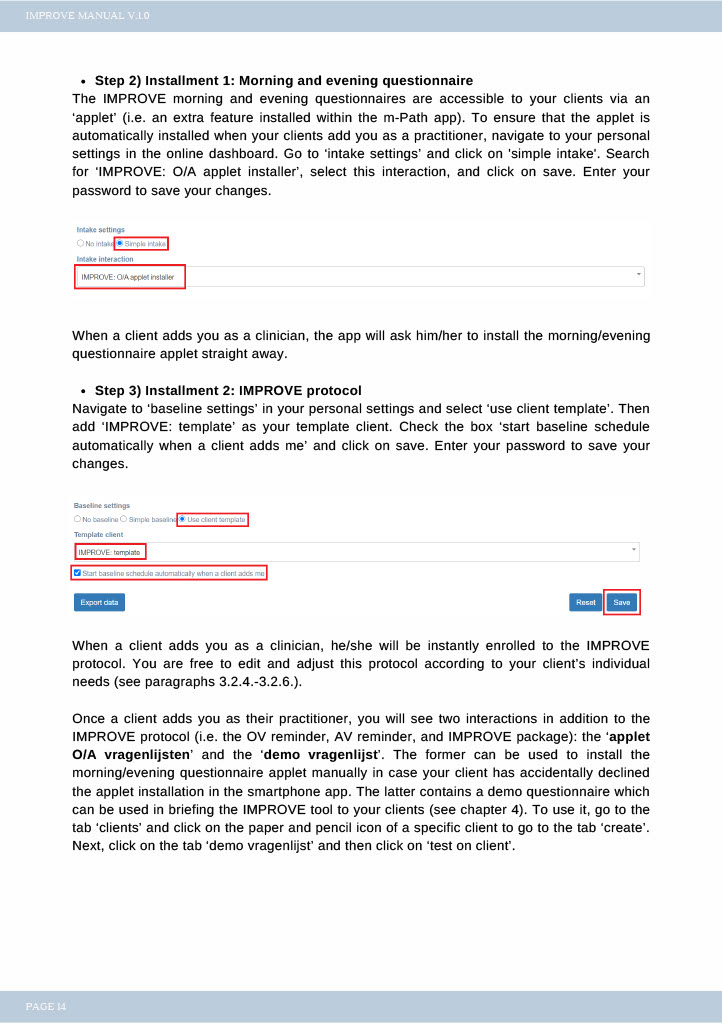

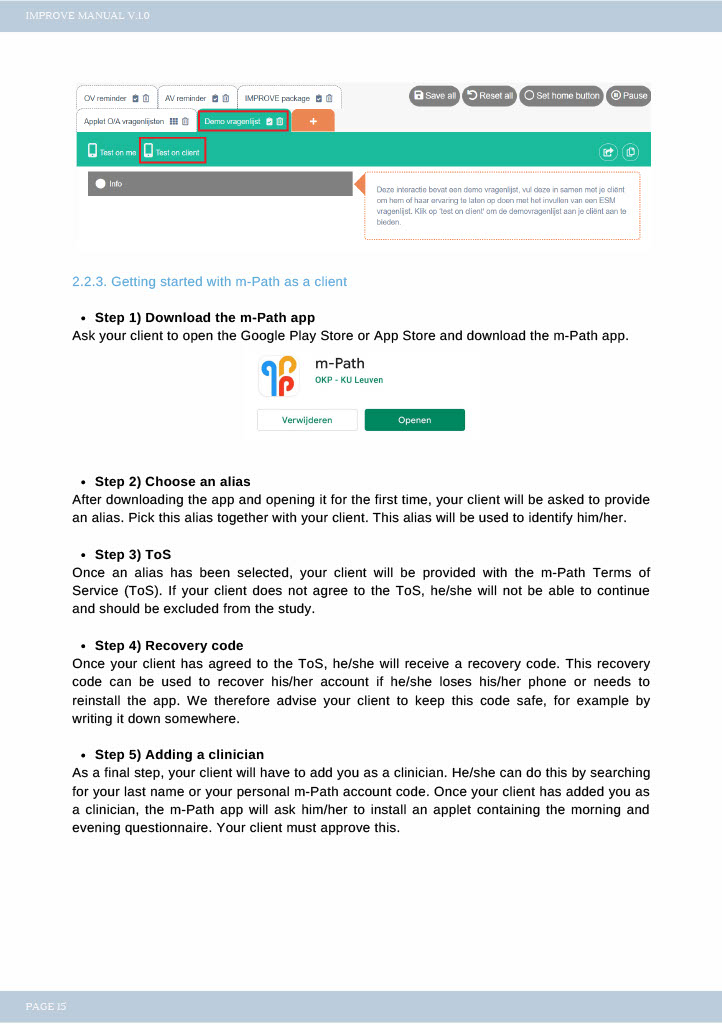

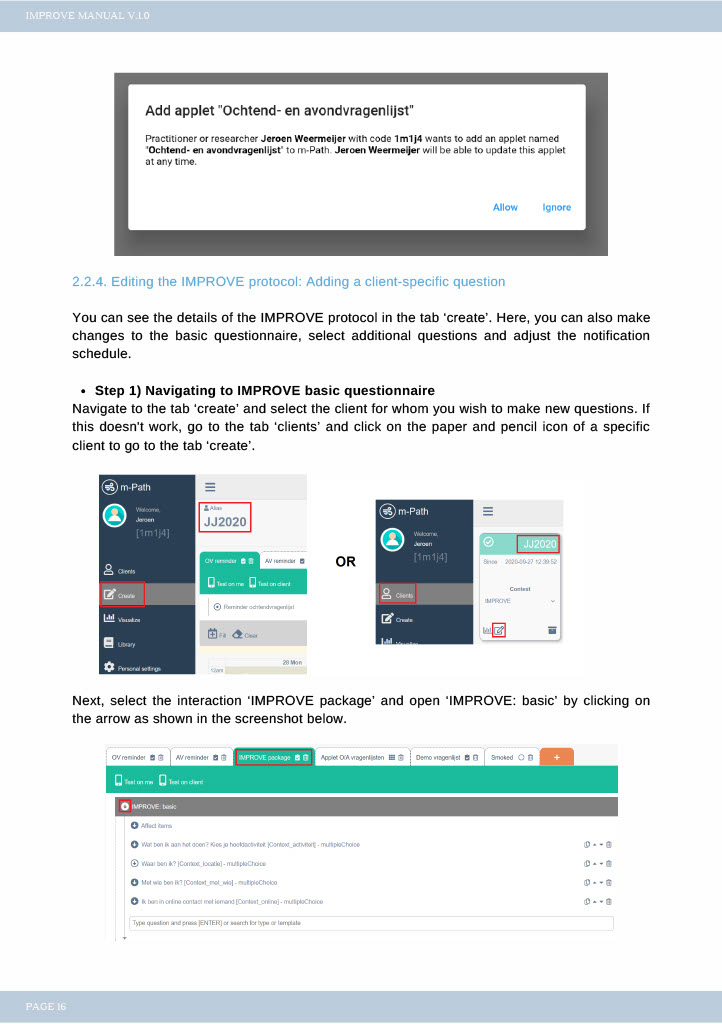

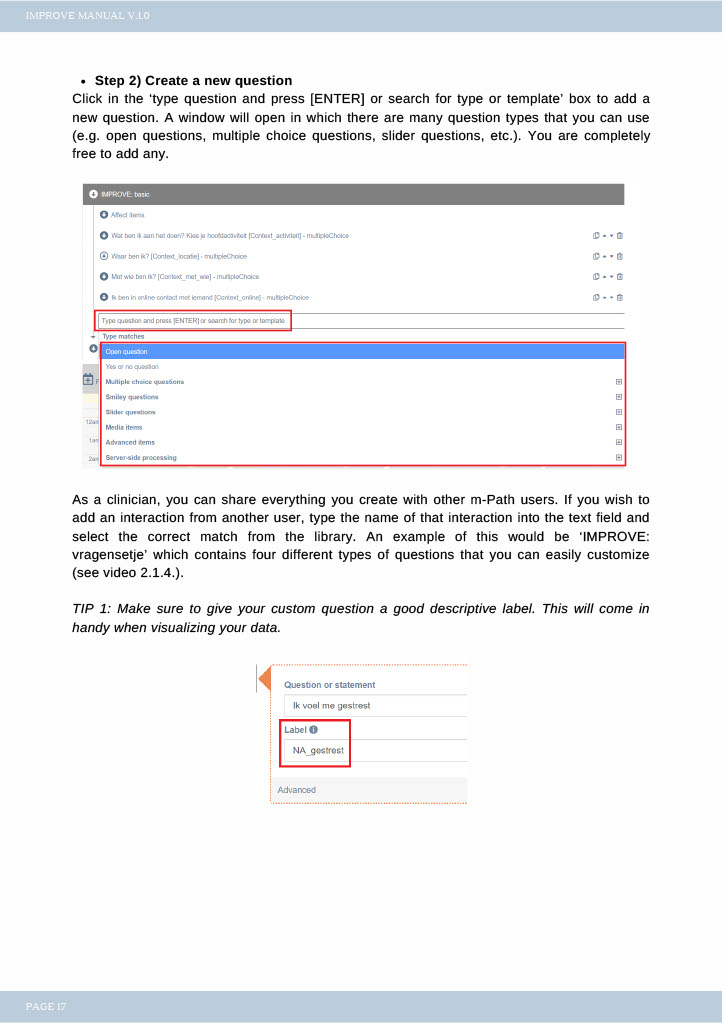

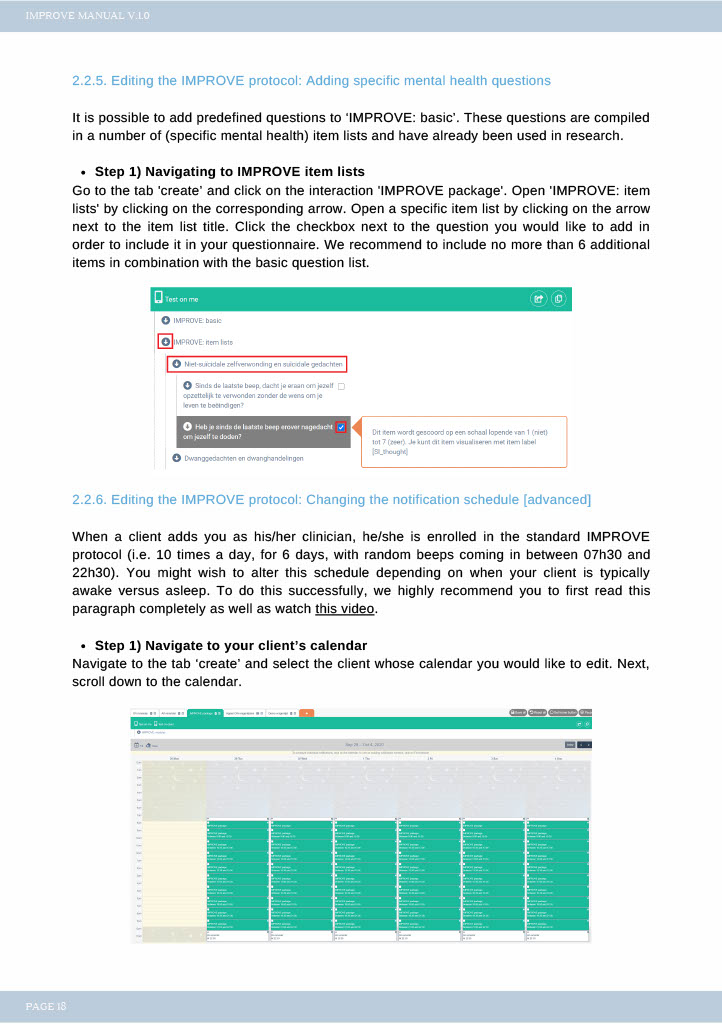

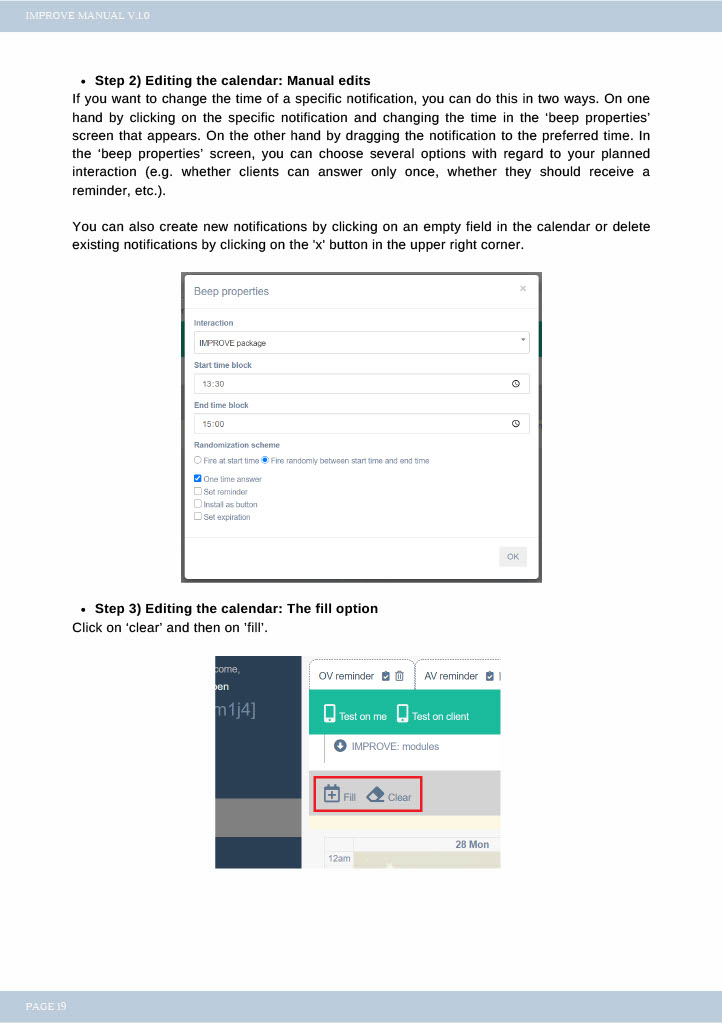

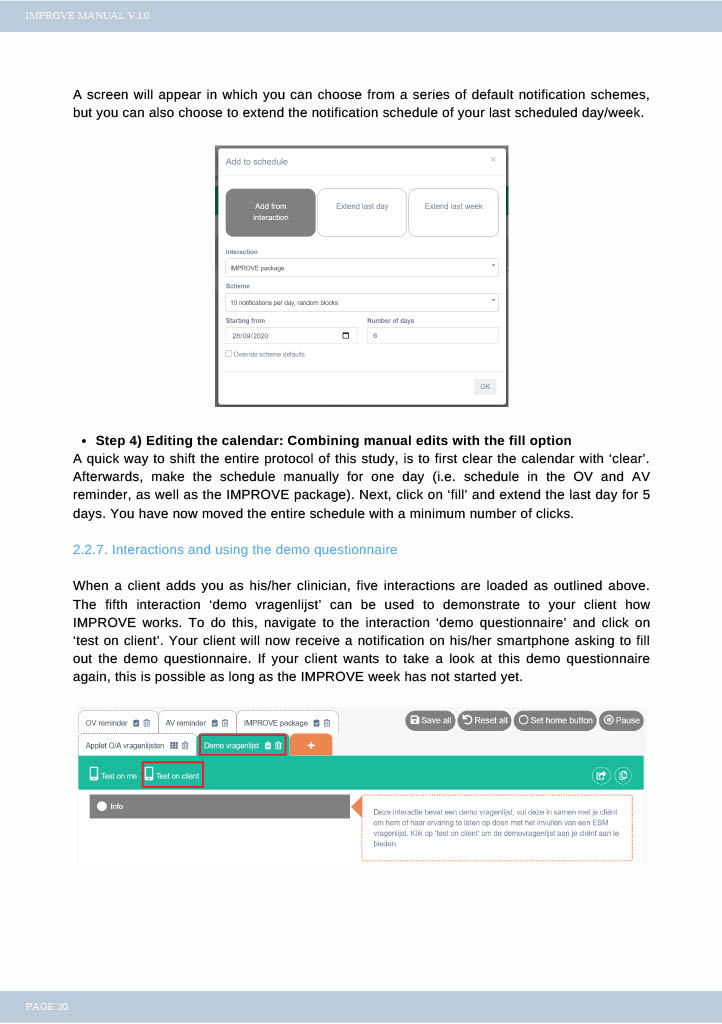

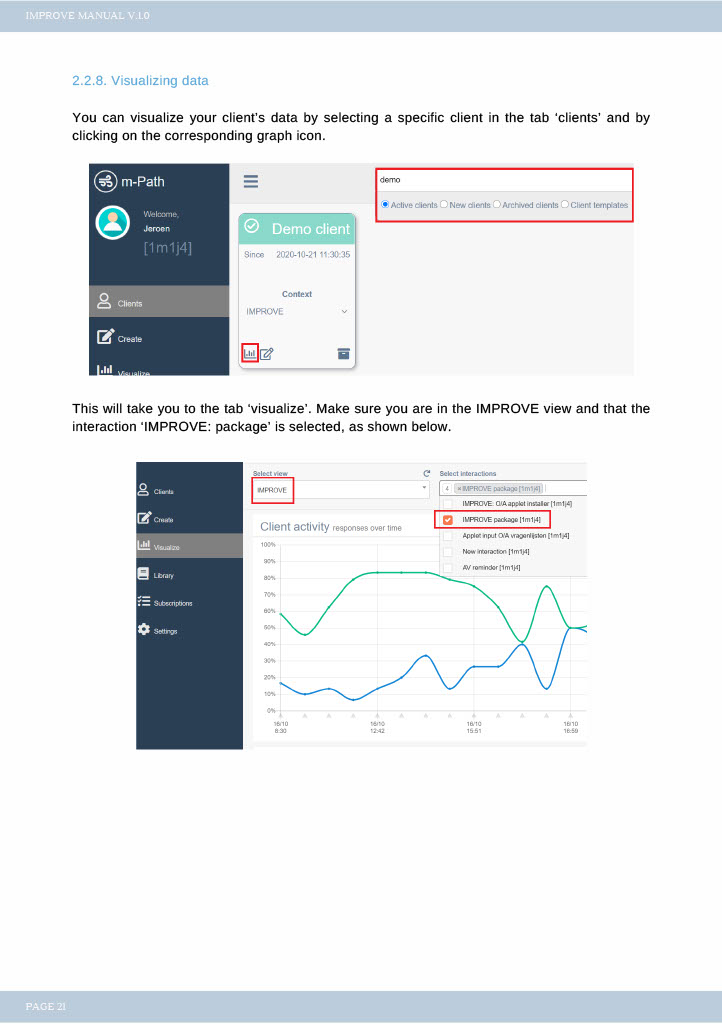

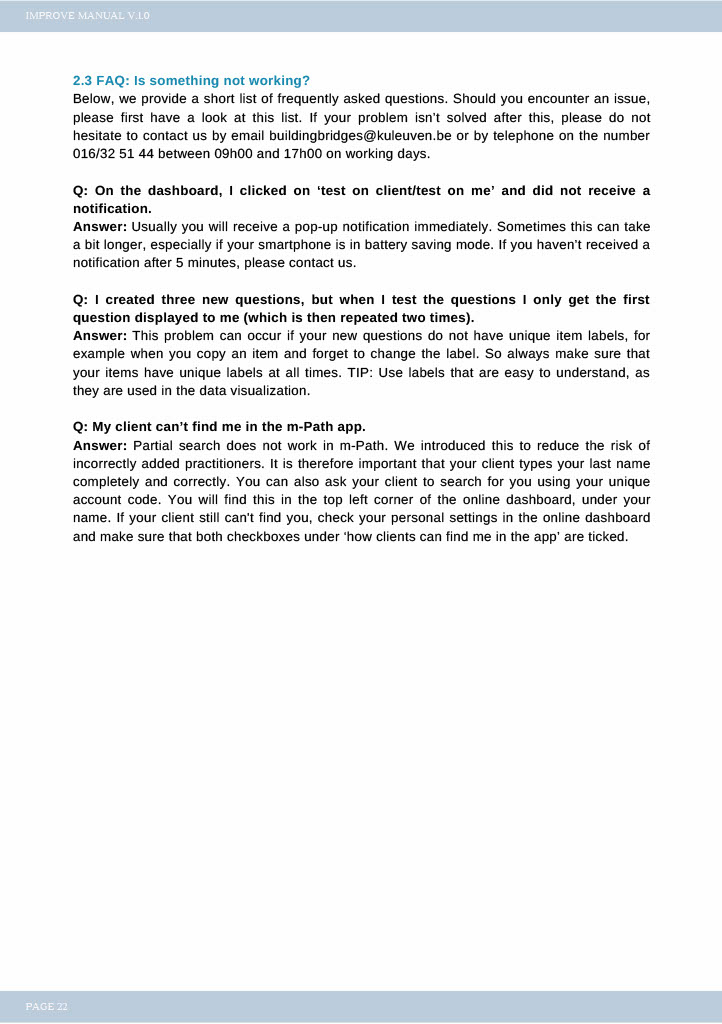

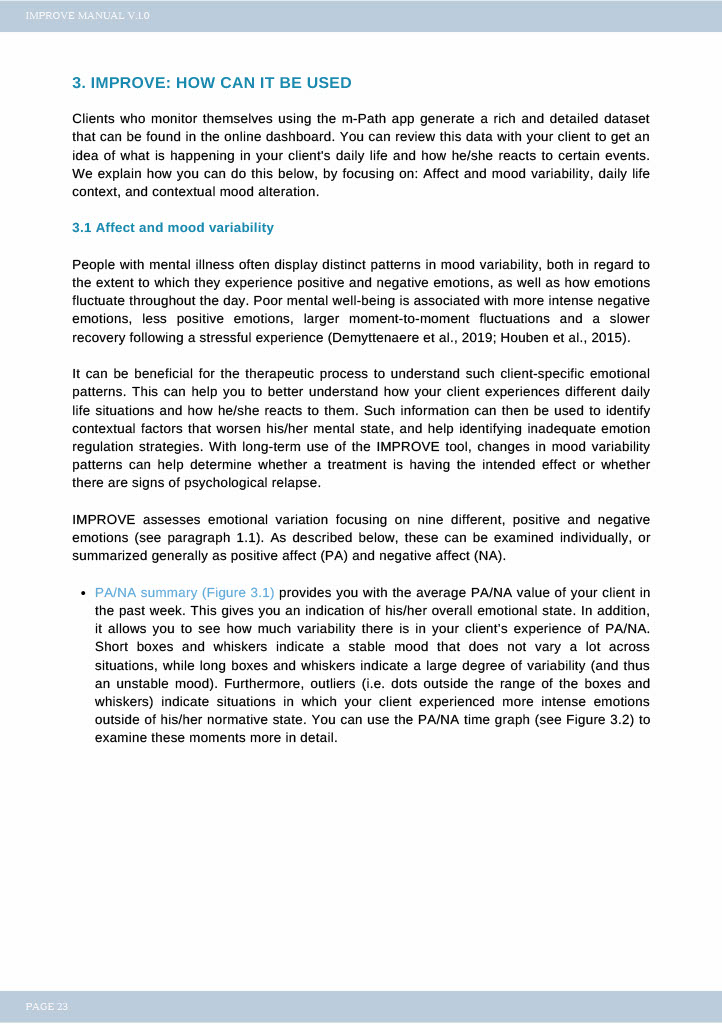

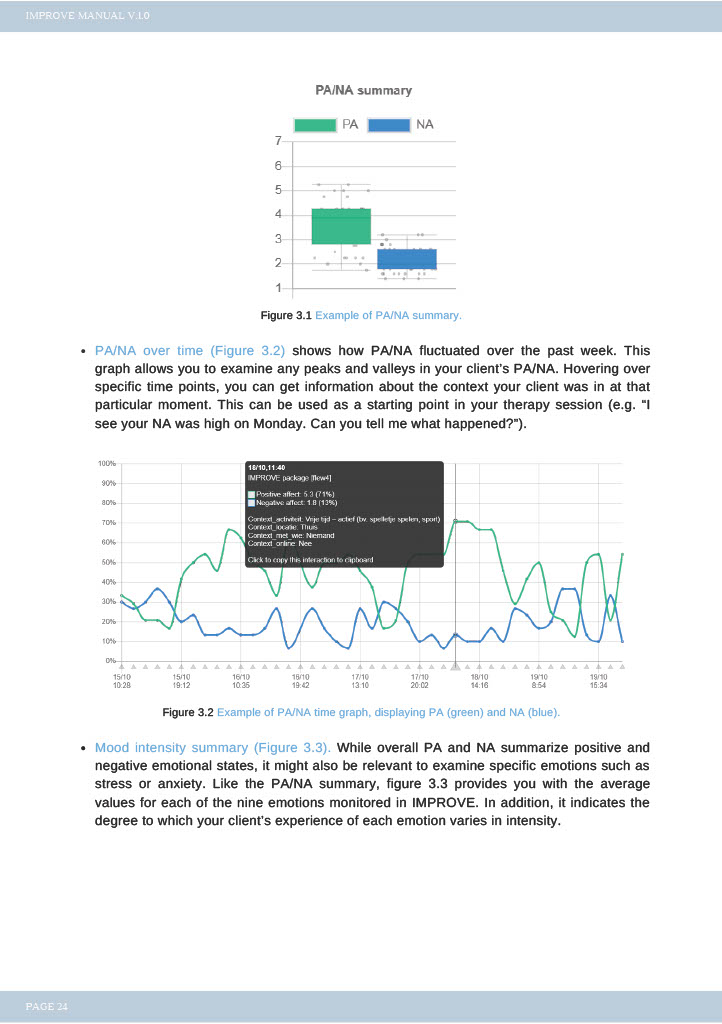

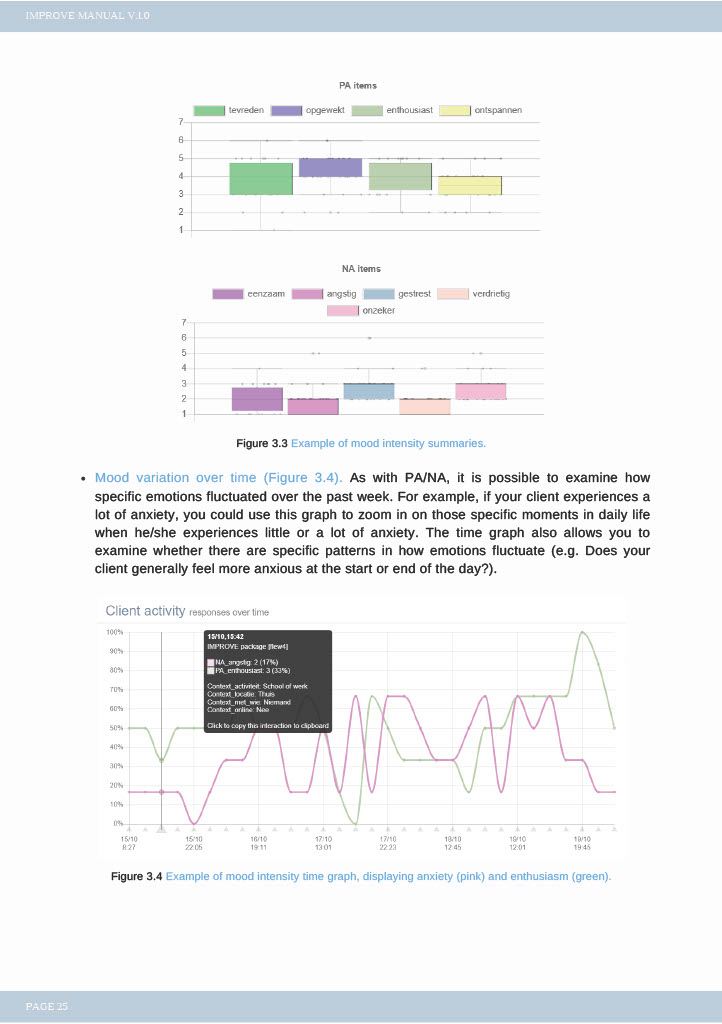

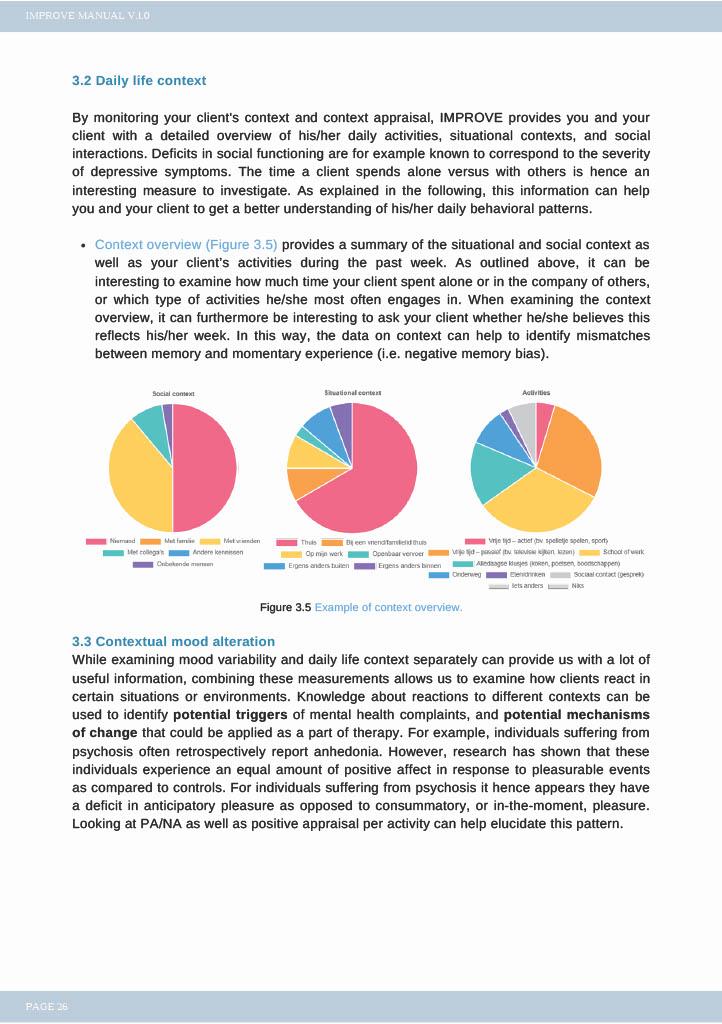

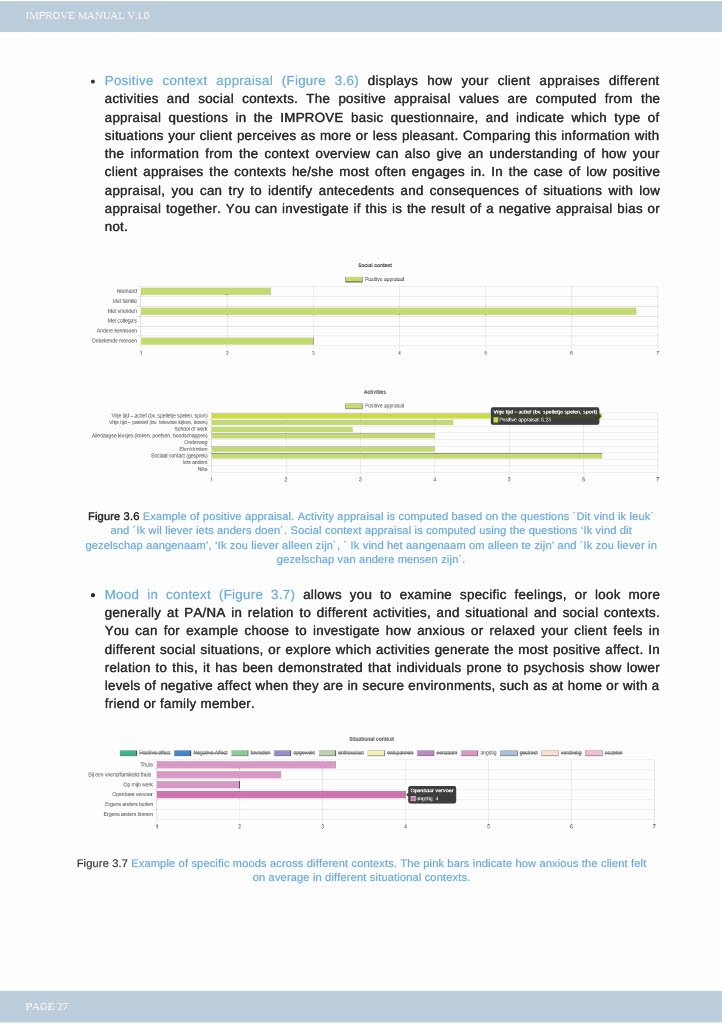

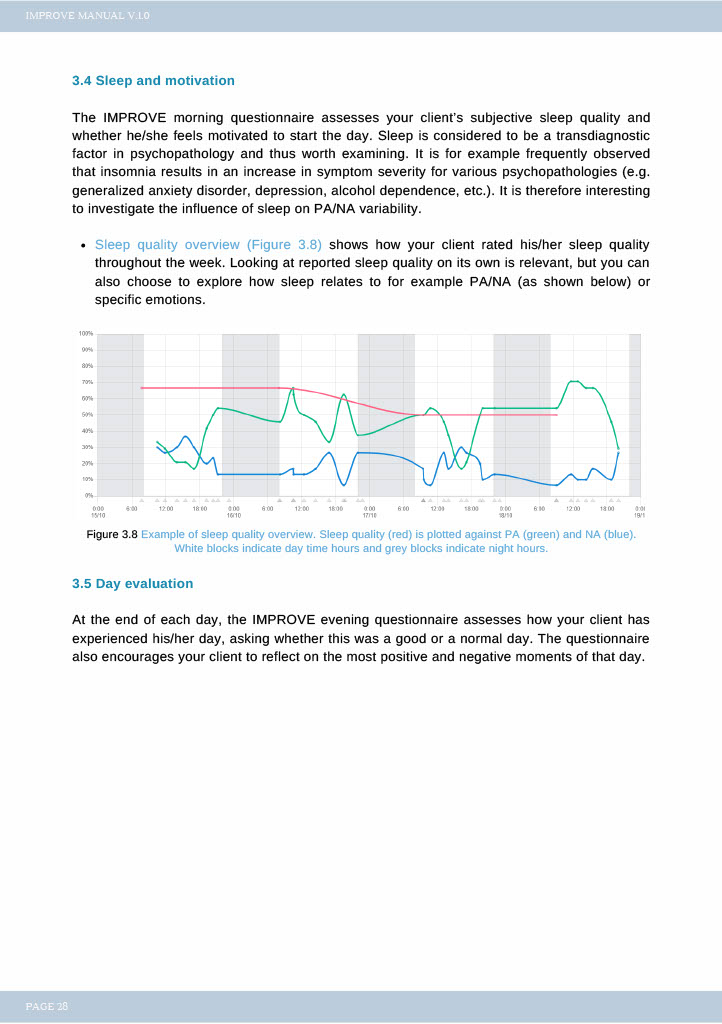

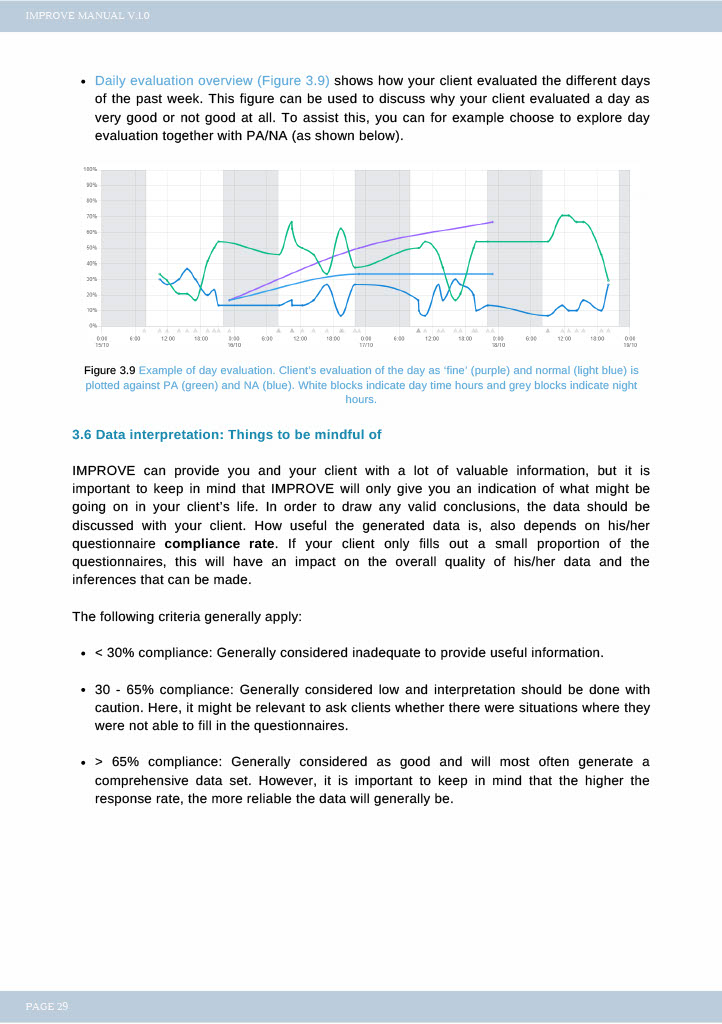

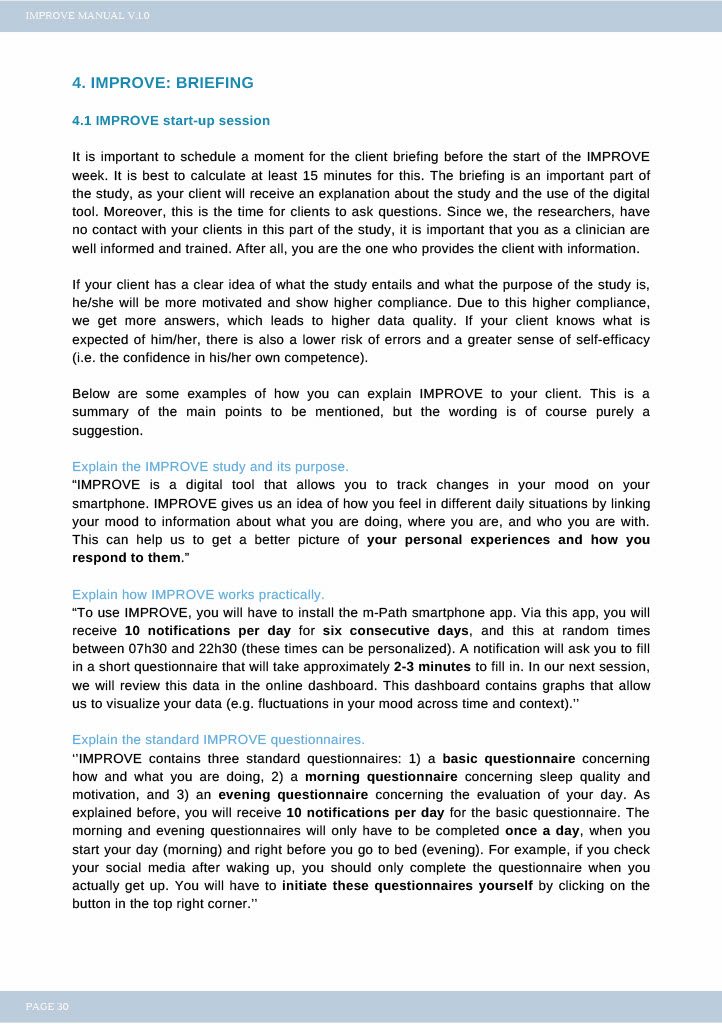

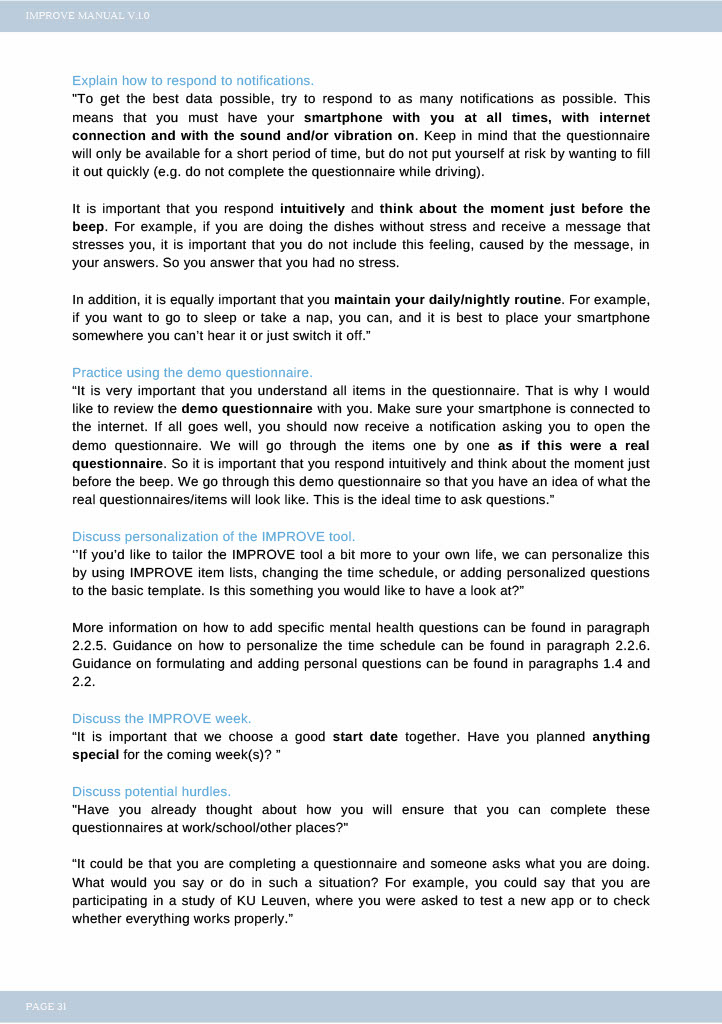

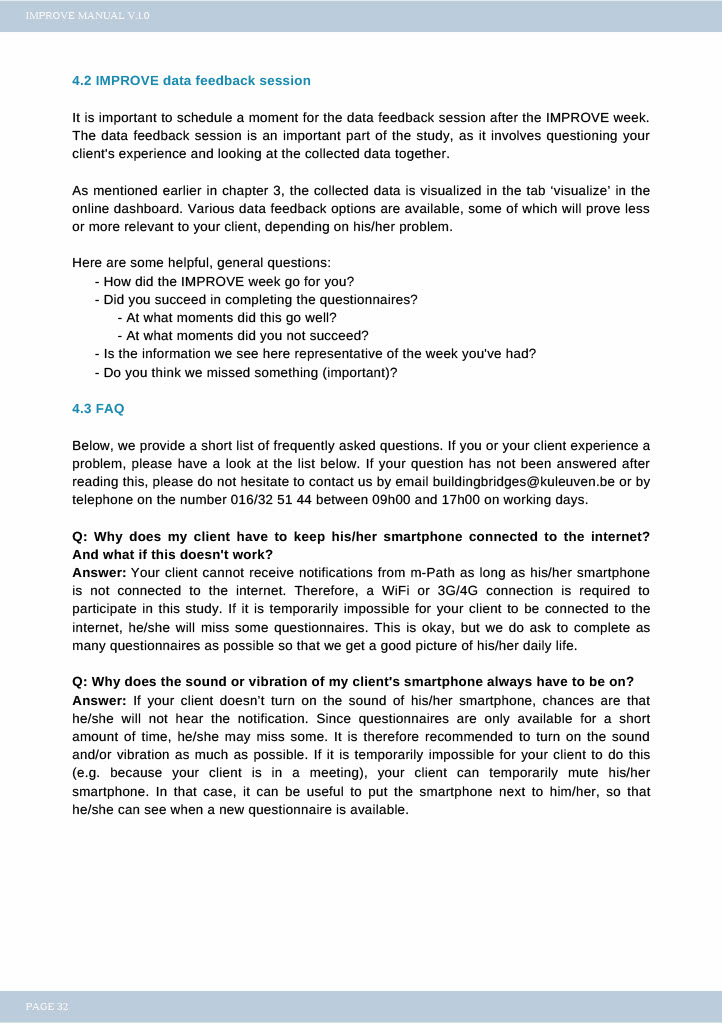

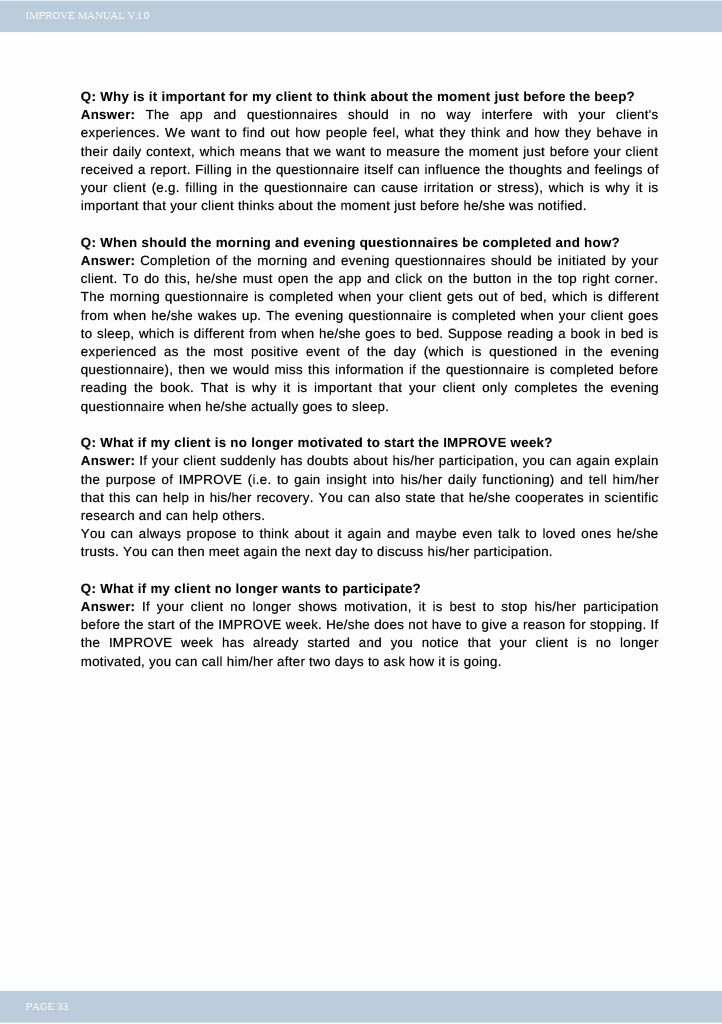

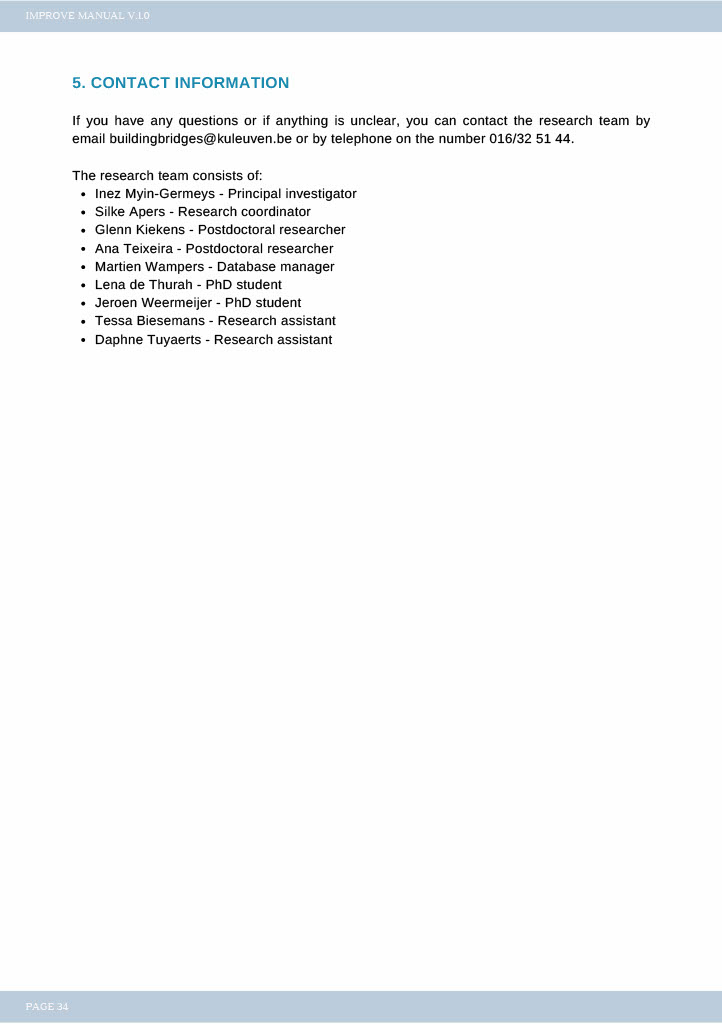

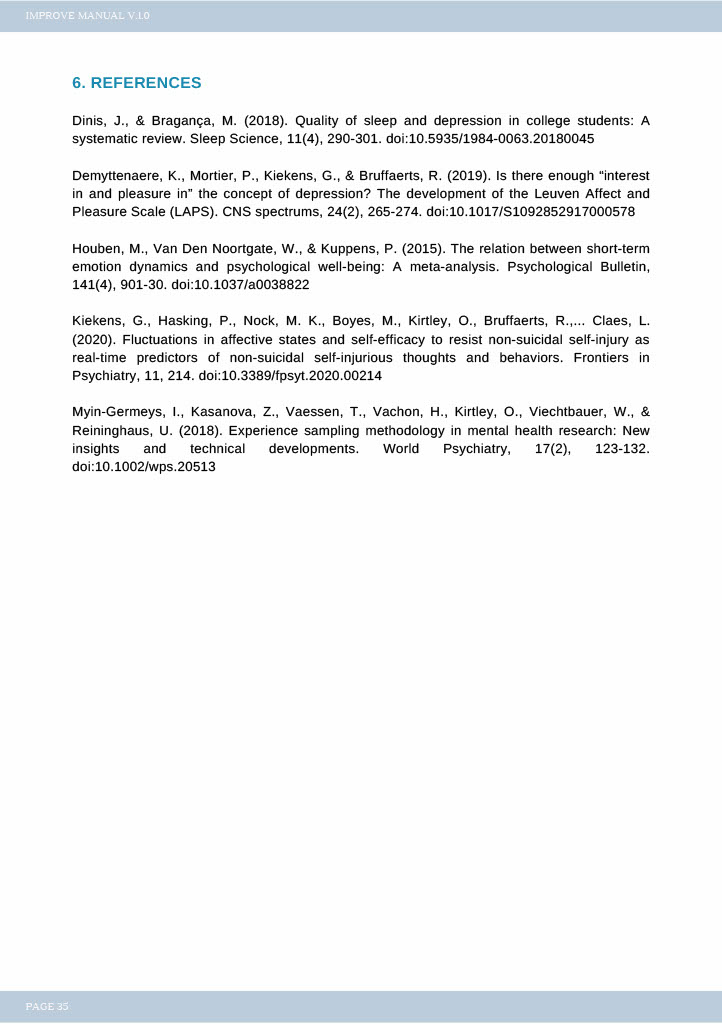

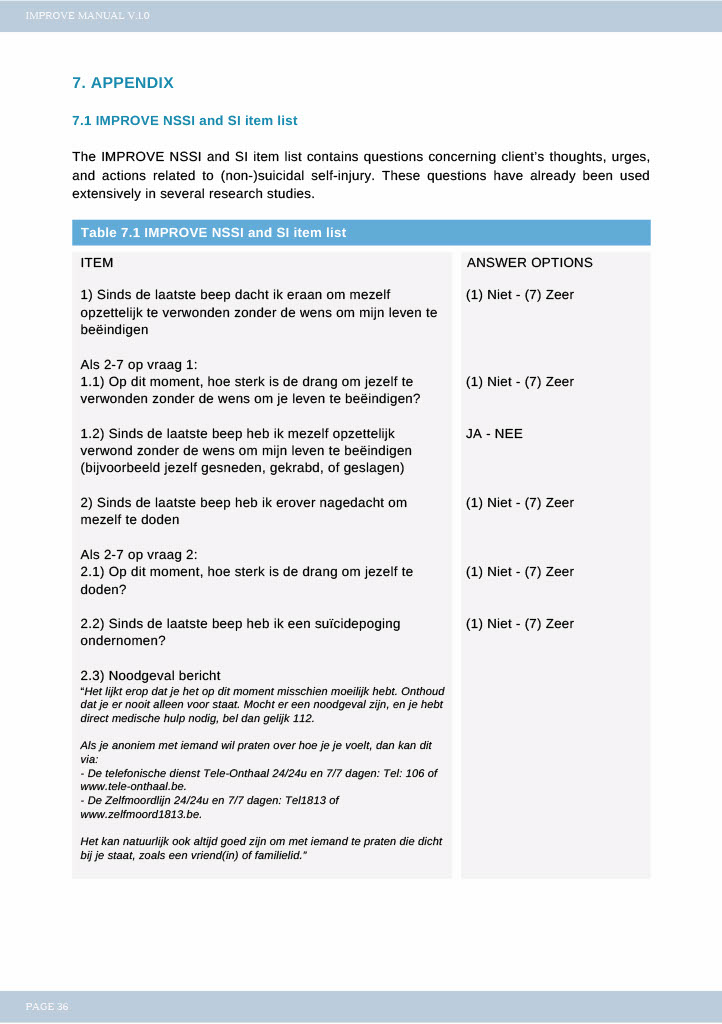
**

Supplement: Multimedia Appendix 1 [file humanfactors-v12-e60096-s001.doc]
