# Supplementary material for: Understanding Appropriation of Digital Self-Monitoring Tools in Mental Health Care: Qualitative Analysis
Source: JMIR Hum Factors. 2025 Mar 3;12:e60096. doi: 10.2196/60096 (PMC11892539; doi:10.2196/60096)
Supplement: Multimedia Appendix 2 [file humanfactors-v12-e60096-s002.doc]

**Multimedia Appendix 2: Interview guides**

IMPROVE: Interviewgids voor behandelaars

**Instructies voor de interviewer**

Deze interviewgids is ontwikkeld om interviewers te ondersteunen in het afnemen van semi-gestructureerde interviews met participanten van de IMPROVE feasibility study. Lees en maak jezelf vertrouwd met de interviewgids alvorens je het interview afneemt!

Deze gids is onderverdeeld in verschillende thematische secties. Elke sectie start met een korte introductie over het onderwerp. Secties bestaan uit open vragen die opgevolgd worden door meer specifieke vragen. Alle vragen dienen aan participanten gesteld te worden, maar als een specifieke vraag al door de participant is beantwoord tijdens het beantwoorden van een open vraag, mag de specifieke vraag niet opnieuw worden gesteld (sla deze over).

Moedig participanten aan om zo veel en vrij mogelijk te spreken, maar zorg ervoor dat er niet (teveel) afgeweken wordt. Als een participant de neiging heeft om uitgebreid te vertellen, stel dan vriendelijk en beleefd voor om naar de volgende vraag te gaan. Probeer participanten niet te onderbreken of antwoorden te suggereren, en zorg ervoor dat je begrijpt wat ze in hun antwoorden bedoelen. Als je het niet zeker weet, vraag hen dan om uitleg.

De interviewer kan ervoor kiezen om kleine wijzigingen aan te brengen in de formulering van de vragen om deze natuurlijker te maken, maar de inhoud en betekenis van de vragen mogen niet gewijzigd worden.

Het interview duurt ongeveer 60 minuten. Houd de tijd bij om er zeker van te zijn dat je deze interviewgids binnen het voorziene tijdsbestek doorloopt.

**Interviewgids**

Introductie en praktische informatie

*Mijn naam is Daphne, ik werk als onderzoeksassistent in het Building Bridges-team, en ik zal vandaag dit interview afnemen.*

*Allereerst* ***bedankt*** *dat je de tijd hebt genomen om met mij te spreken. Het delen van jouw ervaring is erg waardevol voor ons om te begrijpen hoe we onze tool kunnen verbeteren.*

*In dit interview gaan we in op verschillende thema’s. We zullen focussen op jouw eerdere ervaring met en mogelijk toekomstig gebruik van digitale gezondheidstools, jouw mening over deze tool, en hoe het voor je was om deze tool als onderdeel van therapie te gebruiken.*

*De* ***audio*** *van dit interview wordt* ***opgenomen****. De opname zal opgeslagen worden met behulp van een* ***geanonimiseerde studie-ID*** *en zal door een teamlid getranscribeerd worden (d.i. omgezet worden in tekst), die vervolgens gebruikt kan worden voor analyse. Het interview duurt ongeveer* ***60 minuten****. Laat het mij zeker weten als je op een bepaald momenten een* ***pauze*** *wil.*

*Heb je op dit moment nog* ***vragen****? Zo niet, dan start ik het interview en de opname.*

*Je hebt deelgenomen aan de eerste testfase van de IMPROVE-tool, met behulp van het digitale platform (app) m-Path.*

*In dit eerste deel van het interview zou ik graag wat meer te weten willen komen over jouw eerdere ervaringen met dit soort tool.*

**1.** Met welke cliëntenpopulatie werk je? Vanuit welk theoretisch kader werk je gewoonlijk?

**2.** Wat is jouw algemene mening over blended care (d.i. een combinatie van online en face-to-face therapie) en het gebruik van digitale gezondheidstools?

**2.1.** Was dit de eerste keer dat je een digitale gezondheidstool gebruikte in jouw klinisch werk?

**2.2.** Wat waren jouw verwachtingen? Werd hieraan voldaan?

**3.** Wat was jouw kennis van en ervaring met zelf-monitoring door cliënten (bv. experience sampling methods) voordat je aan deze studie deelnam?

**3.1.** Hoe relevant is het volgens jou om zelfmonitoring te implementeren in de GGZ?

*In dit tweede deel van het interview zullen we wat meer focussen op jouw cliënten en hoe het voor je was om deze tool te hebben als onderdeel van de behandeling.*

**4.** Wat maakte dat je IMPROVE introduceerde/voorstelde aan een cliënt?

**4.1.** Waren er cliënten die niet bereid waren de tool te gebruiken? Zo ja, wat heeft hem/haar doen weigeren volgens jou?

**5.** Hoe was het voor jou om de IMPROVE-tool te gebruiken als onderdeel van jouw behandeling?

**5.1**. Wanneer gebruikte je de tool doorgaans (ter voorbereiding van/tijdens sessies)?

**5.2.** Welke functie had de tool in je werk (bv. conversatiestarter)?

*In dit derde deel van het interview zou ik graag wat meer focussen op wat je vindt van de vragen die we gebruikt hebben en de verschillende functies binnen de IMPROVE-tool.*

**6.** Wat vond je van de vragen in de vaste IMPROVE-vragenlijsten (d.i. basis-, ochtend- en avondvragenlijsten)?

**6.1** Vond je ze relevant?

**6.2** Heb je liever andere of aanvullende vragen die voor alle soorten cliënten gebruikt kunnen worden?

**7.** Naast de vaste vragenlijsten, konden sommige functies gepersonaliseerd worden (bv. vragen of antwoordopties toevoegen). Wat vond je daarvan?

**7.1.** Heb je gebruik gemaakt van een van de personalisatie-opties (bv. vragen toevoegen uit IMPROVE itemlijsten, antwoordopties bewerken)? Waarom (niet)?

**7.2.** Hoe belangrijk vind je het (voor jou en jouw cliënten) om gepersonaliseerde opties te hebben?

*In dit volgende deel van het interview zou ik wat meer willen weten over de data feedbacksessies.*

**8.** Hoe was het voor jou om de data met jouw cliënten te bekijken en bespreken?

**8.1.** Waar focuste jij je doorgaans op?

**8.2.** Hadden jij en jouw cliënten gelijkaardige interpretaties van de data?

**8.3.** Wat vond je van de datavisualisaties?

**8.3.1.** Waren ze makkelijk te begrijpen? Zo niet, wat was ingewikkeld?

**8.3.2.** Welke figuren en grafieken vond je het meest nuttig/relevant?

**8.3.3.** Was er informatie waarvan je vond dat deze ontbrak (aanvullende visualisaties die je graag had willen hebben)?

**8.4.** Heeft het gebruik van IMPROVE informatie opgeleverd die is of kan worden gebruikt om nieuwe therapeutische doelen vast te stellen of nieuwe strategieën toe te passen? Kan je een voorbeeld geven?

*We zijn ongeveer halfweg het interview. Ik ga je nu enkele vragen stellen omtrent de impact die het gebruik van de IMPROVE-tool mogelijks gehad heeft op jou en/of jouw cliënten.*

**9.** Welke veranderingen heeft IMPROVE teweeggebracht bij jou en jouw cliënten?

**9.1.** Wat vind je van deze veranderingen (+/-)?

**9.2.** Wie heeft deze veranderingen geïnitieerd?

**9.3.** Heeft het de werkrelatie met jouw cliënten veranderd? Zo ja, hoe?

**9.4.** Heb je het gevoel dat het gebruik van IMPROVE ervoor heeft gezorgd dat jouw cliënten een meer/minder actieve rol opnemen in jouw behandeling?

**9.4.1.** Zijn jouw cliënten meer betrokken bij besluitvormingsprocessen (bv. richting geven aan behandeling) dan voordien?

*Nu zou ik je wat meer willen vragen over hoe het praktisch voor je was om deze tool in jouw werkroutine te hebben.*

**10.** Hoe was het voor jou om de IMPROVE-tool te implementeren in jouw werkroutine?

**10.1.** Hoe was het voor jou om ermee aan de slag te gaan? Makkelijk/moeilijk?

**10.2.** Heb je feedback op het trainingsmateriaal?

**10.3.** Heb je technische problemen ondervonden (bv. verbindingsproblemen)?

**11.** Hoeveel tijd denk je dat je gemiddeld (per sessie) op het dashboard hebt doorgebracht?

**11.1.** Vind je dat een gepaste duur?

**11.2.** Denk je aan veranderingen die we kunnen doorvoeren om het meer tijdsefficiënt te maken?

*We zijn aangekomen bij het laatste deel van het interview. Ik wil het kort even hebben over mogelijk toekomstig gebruik van digitale gezondheidstools en eventuele suggesties voor verbetering.*

**12.** Denk je dat deze tool een meerwaarde heeft voor de cliëntenpopulatie waar je mee werkt? Waarom (niet)?

**13.** Zou je IMPROVE/m-Path (of een gelijkaardige tool) graag willen blijven gebruiken in jouw klinische praktijk? Waarom (niet)?

**14.** Is er iets dat je aan de tool zou veranderen (bv. aanpassen per therapiestroming)? Wat en waarom?

**15.** Is er nog iets dat je zou willen toevoegen?

*Je zal hierna nog een e-mail ontvangen met daarin de link naar een* ***vragenlijst****. Vergeet deze dus zeker niet in te vullen!*

*Nogmaals* ***bedankt*** *voor je tijd. We hopen dat je het leuk vond om aan deze pilot studie deel te nemen. Mocht je vragen hebben over deze studie, dan kan je altijd contact met ons opnemen via ons e-mailadres of telefoonnummer.*

IMPROVE: Interviewgids voor cliënten

**Instructies voor de interviewer**

Deze interviewgids is ontwikkeld om interviewers te ondersteunen in het afnemen van semi-gestructureerde interviews met participanten van de IMPROVE feasibility study. Lees en maak jezelf vertrouwd met de interviewgids alvorens je het interview afneemt!

Deze gids is onderverdeeld in verschillende thematische secties. Elke sectie start met een korte introductie over het onderwerp. Secties bestaan uit open vragen die opgevolgd worden door meer specifieke vragen. Alle vragen dienen aan participanten gesteld te worden, maar als een specifieke vraag al door de participant is beantwoord tijdens het beantwoorden van een open vraag, mag de specifieke vraag niet opnieuw worden gesteld (sla deze over).

Moedig participanten aan om zo veel en vrij mogelijk te spreken, maar zorg ervoor dat er niet (teveel) afgeweken wordt. Als een participant de neiging heeft om uitgebreid te vertellen, stel dan vriendelijk en beleefd voor om naar de volgende vraag te gaan. Probeer participanten niet te onderbreken of antwoorden te suggereren, en zorg ervoor dat je begrijpt wat ze in hun antwoorden bedoelen. Als je het niet zeker weet, vraag hen dan om uitleg.

De interviewer kan ervoor kiezen om kleine wijzigingen aan te brengen in de formulering van de vragen om deze natuurlijker te maken, maar de inhoud en betekenis van de vragen mogen niet gewijzigd worden.

Het interview duurt ongeveer 60 minuten. Houd de tijd bij om er zeker van te zijn dat je deze interviewgids binnen het voorziene tijdsbestek doorloopt.

**Interviewgids**

Introductie en praktische informatie

*Mijn naam is Daphne, ik werk als onderzoeksassistent in het Building Bridges-team, en ik zal vandaag dit interview afnemen.*

*Allereerst* ***bedankt*** *dat je de tijd hebt genomen om met mij te spreken. Het delen van jouw ervaring is erg waardevol voor ons om te begrijpen hoe we onze tool kunnen verbeteren.*

*In dit interview gaan we in op verschillende thema’s. We zullen focussen op jouw eerdere ervaring met en mogelijk toekomstig gebruik van digitale gezondheidsapps, jouw mening over deze tool, hoe het voor je was om deze te gebruiken, en de impact die het mogelijks gehad heeft op jou(w behandeling) en jouw dagelijks leven.*

*De* ***audio*** *van dit interview wordt* ***opgenomen****. De opname zal opgeslagen worden met behulp van een* ***geanonimiseerde studie-ID*** *en zal door een teamlid getranscribeerd worden (d.i. omgezet worden in tekst), die vervolgens gebruikt kan worden voor analyse. Het interview duurt ongeveer* ***60 minuten****. Laat het mij zeker weten als je op een bepaald momenten een* ***pauze*** *wil.*

*Heb je op dit moment nog* ***vragen****? Zo niet, dan start ik het interview en de opname.*

*Je hebt deelgenomen aan de eerste testfase van de IMPROVE-tool, gebruik makende van de m-Path app.*

*In dit eerste deel van het interview zou ik graag wat meer te weten willen komen over jouw eerdere ervaringen met digitale gezondheidsapps, alsook waarom je hebt deelgenomen aan deze studie.*

**1.** Hoe lang ben je al in behandeling bij je huidige behandelaar?

**2.** Wat dacht je toen jouw behandelaar voorstelde om aan deze studie deel te nemen?

**2.1.** Waarom nam je deel?

**2.2.** Wat waren jouw persoonlijke verwachtingen?

**3**. Heb je eerder zelfcontrole-apps (of andere apps voor geestelijke gezondheid) gebruikt? Waarom (niet)? Zo ja, gebruik je die app(s) nu nog? Indien nee, waarom ben je ermee gestopt?

*In dit tweede deel van het interview zou ik graag wat meer willen weten over jouw ervaring met de m-Path app en jouw mening over de vragenlijsten die je bij elke piep hebt ingevuld.*

**4.** Hoe was het voor jou om de m-Path app te gebruiken?

**4.1.** Hoe was het voor jou om meerdere keren per dag aandacht te (moeten) schenken aan je gevoelens?

**5.** Wat vond je van de vragen die je in de app voorgelegd kreeg?

**5.1.** Vond je ze relevant?

**5.2.** Waren ze gemakkelijk te begrijpen en te beantwoorden?

**6.** Jij en jouw behandelaar hadden de mogelijkheid om de vragenlijsten te personaliseren (bv. vragen of antwoordopties toevoegen). Heb je dit met jouw behandelaar besproken?

**6.1.** Heb je gebruik gemaakt van een van de personalisatie-opties (bv. vragen toevoegen uit IMPROVE itemlijsten, antwoordopties bewerken)? Waarom (niet)?

**6.2.** Hoe belangrijk vind je het om gepersonaliseerde opties te hebben?

**7.** Is er nog iets dat je graag gemeten had met betrekking tot jouw geestelijke gezondheid?

**8.** Wat vond je van de frequentie (d.i. het aantal notificaties)?

**8.1.** Wat zou het ideale aantal notificaties zijn?

**8.2.** Op hoeveel van de notificaties denk je dat je per dag hebt gereageerd?

**9.** Hoe lang duurde het gewoonlijk om de vragenlijsten in te vullen?

**9.1.** Vond je dat aanvaardbaar?

**9.2.** Wat zou de ideale hoeveelheid tijd zijn om vragen te beantwoorden?

**10.** Hoe paste het gebruik van de app in je dagelijkse routine?

**10.1.** Heb je iets in jouw dagelijkse leven veranderd (bv. routine, levenscontexten) om op notificaties te kunnen reageren?

**10.2.** Was het ooit ongepast/lastig voor jou om op notificaties te reageren?

**11.** Heb je technische problemen ondervonden tijdens het gebruik van de app (bv. verbindingsproblemen)?

*We zitten nu in het derde deel van het interview. Het idee was dat je na jouw zelfmonitoring week met jouw behandelaar zou samenkomen om jouw data te bespreken.*

**12.** Hebben jullie tijd besteed aan het bekijken van jouw data?

*Ok, goed. Ik ga je nu wat vragen stellen omtrent de mogelijkheden die IMPROVE biedt met betrekking tot datafeedback.*

**12.1.** Hoe was het voor jou om jouw data met jouw behandelaar te bekijken en bespreken?

**12.1.1.** Waar focuste jij je doorgaans op?

**12.1.2.** Hadden jij en jouw behandelaar gelijkaardige interpretaties van de data?

**12.1.3.** Hoe was het voor jou om je data gevisualiseerd te zien op het dashboard?

**12.1.3.1.** Weerspiegelden de figuren en grafieken nauwkeurig de week die je had?

**12.1.4.** Wat vond je van de figuren en grafieken die gebruikt werden om je data te visualiseren?

**12.1.4.1.** Waren ze makkelijk te begrijpen? Zo niet, wat was ingewikkeld?

**12.1.4.2.** Welke figuren en grafieken vond je het meest nuttig/relevant?

*We zijn halverwege het interview. Ik ga je nu enkele vragen stellen over de impact die het gebruik van de IMPROVE-tool mogelijks gehad heeft op jou(w behandeling) en/of jouw behandelaar.*

**13.** Heb je het gevoel dat je iets nieuws hebt geleerd over jezelf/jouw geestelijke gezondheid? Zo ja, was het de dagelijkse zelfmonitoring of het bespreken van de data met jouw behandelaar die je heeft geholpen om tot deze nieuwe inzichten te komen?

**14.** Heeft het gebruik van IMPROVE informatie opgeleverd die jij of jouw behandelaar gebruikt hebben om nieuwe therapeutische doelen vast te stellen of nieuwe strategieën toe te passen?

**15.** Welke veranderingen heeft IMPROVE teweeggebracht voor jouw behandeling?

**15.1.** Wat vind je van deze veranderingen (+/-)?

**15.2.** Wie heeft deze veranderingen geïnitieerd?

**15.3.** Heeft het de werkrelatie met jouw behandelaar veranderd? Zo ja, hoe?

**15.4.** Heb je het gevoel dat het gebruik van IMPROVE ervoor heeft gezorgd dat je een meer/minder actieve rol opneemt in jouw behandeling?

**15.4.1.** Ben je meer betrokken bij besluitvormingsprocessen (bv. richting geven aan behandeling) dan voordien?

*Ik zou wat meer willen weten over de impact die het gebruik van de IMPROVE-tool mogelijks gehad heeft op jouw dagelijks leven.*

**16.** Heb je iets veranderd (of denk je eraan om iets te veranderen) aan jouw dagelijkse routine/gedrag, gebaseerd op wat je over jezelf geleerd hebt? Zo ja, wat waren de effecten hiervan?

**17.** Denk je dat je, na het gebruik van IMPROVE, anders/beter met jouw symptomen zal omgaan? Zo ja, hoe?

**18.** Denk je dat je, na het gebruik van IMPROVE, problemen/uitdagingen anders/beter zal oplossen? Zo ja, kan je een voorbeeld geven?

*We zijn aangekomen bij het laatste deel van het interview. Ik wil het kort even hebben over mogelijk toekomstig gebruik van digitale gezondheidsapps en eventuele suggesties voor verbetering.*

**19.** Vind je dat deze tool een toegevoegde waarde heeft voor jou? Waarom (niet)?

**20.** Zou je m-Path (of een gelijkaardige zelfmonitoring app) graag willen blijven gebruiken in de toekomst? Waarom (niet)? Zo ja, wil je de app dan zelfstandig gebruiken of integreer je deze liever in jouw behandeling?

**21**. Is er iets dat je aan de tool zou veranderen? Wat en waarom?

**22.** Is er nog iets dat je zou willen toevoegen?

*Nogmaals* ***bedankt*** *voor je tijd. We hopen dat je het leuk vond om aan deze pilot studie deel te nemen. Mocht je vragen hebben over deze studie, dan kan je altijd contact met ons opnemen via ons e-mailadres of telefoonnummer.*
