# Supplementary material for: Understanding Appropriation of Digital Self-Monitoring Tools in Mental Health Care: Qualitative Analysis
Source: JMIR Hum Factors. 2025 Mar 3;12:e60096. doi: 10.2196/60096 (PMC11892539; doi:10.2196/60096)
Supplement: Multimedia Appendix 3 [file humanfactors-v12-e60096-s003.doc]

**Multimedia appendix 3: Demographic summary**

|  | **Clinicians (n=7)** | **Clients (n = 11)** |
| --- | --- | --- |
| **Age, mean [range]** | 45 years [34-51] | 37 years [19-58] |
| **Work experience in mental health care, mean [range]** | 14 years [5-26] |  |
| **Gender**   - Female - Male | 6 (86%)  1 (14%) | 8 (73%)  3 (27%) |
| **Profession**   - Psychologist - Nurse - Other health profession | 4 (57%)  2 (29%)  1 (14%) |  |
| **Self-reported diagnosis**   - Addiction - ADHD - Anxiety - Bipolar disorder - Burnout - Depression - Eating disorder - None - OCD - Other - Psychosis |  | 1 (9%)  2 (18%)  4 (36%)  1 (9%)  2 (18%)  4 (36%)  4 (36%)  2 (18%)  2 (18%)  1 (9%)  2 (18%) |
