# Supplementary material for: Understanding Appropriation of Digital Self-Monitoring Tools in Mental Health Care: Qualitative Analysis
Source: JMIR Hum Factors. 2025 Mar 3;12:e60096. doi: 10.2196/60096 (PMC11892539; doi:10.2196/60096)
Supplement: Multimedia Appendix 4 [file humanfactors-v12-e60096-s004.doc]

**Multimedia Appendix 4: Overview of themes**

| THEMES AND SUB-THEMES | Mentioned by participants | |
| --- | --- | --- |
|  | **Clinicians (n=7)** | **Clients**  **(n=11)** |
| 1. PRIOR KNOWLEDGE AND EXPECTATIONS | | |
| 1.1. PRIOR EXPERIENCE | **7** | **11** |
| Experience with analog self-monitoring | 6 | 2 |
| Experience with using digital mental health tools | 4 | 4 |
| No experience with digital self-monitoring | 6 | 10 |
| 1.2. EXPECTATIONS | **7** | **11** |
| Beneficial | 7 | 4 |
| Concerns and limitations | 6 | 3 |
| Unclear | 4 | 10 |
| 1.3. MOTIVATION | **6** | **9** |
| Curiosity | 3 | 7 |
| Need to have and learn about digital tools | 5 | 1 |
| Wanting to help research | - | 8 |
| 2. ACTUAL USE IN PRACTICE | | |
| Step 1: *Start-up session* | | |
| 2.1. SYSTEM COMPLEXITY, USER COMPETENCIES AND NEED FOR SUPPORT | **7** | **-** |
| Need for in-person support | 5 | - |
| System complexity too high | 5 | - |
| Consulting training manual for help | 3 | - |
| User competences inadequate | 5 | - |
| 2.2. TIME INVESTMENT AND PRACTICE | **7** | **-** |
| Practice makes perfect | 6 | - |
| Time investment too high | 7 | - |
| 2.3. MAKING USE OF PERSONALIZATION | **7** | **11** |
| Personalization is beneficial | 7 | 9 |
| Personalization requires extra time and effort | 5 | - |
| Personalization was used | 5 | 5 |
| Personalization was not discussed with client | - | 9 |
| *Step 2: Self-monitoring* | | |
| 2.4. RESPONDING TO NOTIFICATIONS | **-** | **11** |
| Assessment frequency and time spent | - | 11 |
| Being motivated to respond | - | 8 |
| Compliance challenges | - | 11 |
| 2.5. ASSESSMENT QUESTIONS | **-** | **6** |
| Open questions and more details | - | 6 |
| 2.6. SELF-REFLECTION | **-** | **6** |
| Negative emotions or self-judgement | - | 4 |
| Difficult labeling and scoring emotion | - | 4 |
| 2.7 REAL-TIME MONITORING OF CLIENT COMPLIANCE | **3** | **-** |
| *Step 3: Data feedback session* | | |
| 2.8. PREPARING FOR SESSION IS NECESSARY | **6** | **-** |
| 2.9. INTERPRETING GRAPHS AND USING DATA | **7** | **11** |
| Difficult understanding and navigating graphs | 6 |  |
| Difficulties interpreting data | 4 | 5 |
| Using only part of the data | 6 | 3 |
| 2.10. REVIEWING DATA TOGETHER | **7** | **11** |
| Co-interpretation | 3 | 2 |
| Reviewed data together | 7 | 8 |
| 3. POTENTIAL FUTURE USE | | |
| 3.1. ADDED VALUE | **6** | **6** |
| More details and information | 6 | 2 |
| Overview, focus and clarity | 5 | 2 |
| Positive overall | 6 | 1 |
| 3.2. ADDITIONAL POTENTIAL | **6** | **6** |
| Measuring other client-specific aspects | 6 | 3 |
| Monitoring progress and treatment effect | 5 | 3 |
| 3.3. FUTURE USE | **7** | **10** |
| Likely to use it again | 3 | 4 |
| Might use it again | 7 | 7 |
| Not likely to use it again | - | 3 |
