# Supplementary material for: Understanding Appropriation of Digital Self-Monitoring Tools in Mental Health Care: Qualitative Analysis
Source: JMIR Hum Factors. 2025 Mar 3;12:e60096. doi: 10.2196/60096 (PMC11892539; doi:10.2196/60096)
Supplement: Multimedia Appendix 5 [file humanfactors-v12-e60096-s005.doc]

**Multimedia Appendix 5: Individually summarized experiences**

Participants’ individual experiences, summarized using in-vivo coding from interviews.

| **PARTICIPANT** | **PRIOR KNOWLEDGE AND EXPECTATIONS** | **ACTUAL USE IN PRACTICE** | **POTENTIAL FUTURE USE** |
| --- | --- | --- | --- |
| **CLINICIAN**  **ID: 0100**  *(Age unknown)*  Female  Psychologist | **EXPERIENCE** - No experience with digital self-monitoring - Currently testing VR technology in therapy - I've previously worked with analog self-registration **EXPECTATION** - I expected it would be technically challenging - I was eager to try it **MOTIVATION** - I want to try using new methods in therapy | **START-UP SESSION** - It was difficult to find things in the dashboard - I needed help from the research team to use the tool - After a while using the platform gets easier, but it requires a lot of time to get started **FEEDBACK SESSION** - It was difficult to draw conclusions about my clients' data I asked my client for clarifications when I was not able to interpret their data - We used the tool to draw conclusions about my clients' complaints | **ADDED VALUE**  - The tool gives you more information about what happens in clients' lives  **FUTURE USE** - I might use the tool again if it is made easier to use. |
| **CLIENT**  **ID: 0102**  58 years old  Male  Self-reported diagnose: anxiety, depression, OCD, burn-out | **EXPERIENCE**  - No prior experience with health apps  **MOTIVATION** - I was curious to see if I would get something out of it  - Wanted to help | **START-UP SESSION** - It would be helpful to go over questions beforehand - It would be helpful if clinicians tried the tool themselves **SELF-MONITORING** - Assessment frequency is unnecessarily high - Assessment can be disturbing - Notifications made me more aware of my phone - I sometimes felt guilty when I missed notifications - I sometimes put off responding to notifications, because there we so many - The questions were not suited to capture my experience - I would have linked more open assessments that allowed me to give more detail - Self-reflection enforced negative self-judgment - Self-reflection was difficult, I was unsure if I was answering the questions correctly.  **DATA FEEDBACK SESSION** - My therapist could make useful conclusions - Interpretation of data was somewhat difficult. |  |
| **CLINICIAN ID: 0300**  34 years old  Female  Psychologist | **EXPERIENCE** - I previously tried using online mental health platforms - I have worked with analog self-registration in therapy **EXPECTATIONS** - Digital tools can be helpful for some but not for everyone - I expected it would be easier to use than analog methods - I was eager to try it but kept my expectations low **MOTIVATION** - Digital technologies have become a big part of our lives - I wanted to give it a try | **START-UP SESSION** - I needed help from the research team to use the tool - I didn't have enough time to familiarize myself with the tool, which was stressful. - I just tried out things **FEEDBACK SESSION** - There is a lot of data, which makes it difficult to know how to conclude.  - I found it difficult to interpret the data. - Choose to look at the simpler graphs. - We examined clients' affect. | **ADDED VALUE**  - The data allows you to examine the clients' story, and help them tell it.  **FUTURE USE** - I will consider using the tool again. |
| **CLIENT**  **ID: 0501**  *(Age unknown)*  Female  Self-reported diagnosis: eating disorder | **EXPERIENCE**  - No experience with self-monitoring app **EXPECTATIONS** - Was hesitant towards participating, does not like self-assessments **MOTIVATION** - I thought it was worth trying - Want to contribute to improve mental health care | **START-UP SESSION** - It was difficult always to remember to have my phone with me - Notifications were sometimes disturbing - Assessment frequency was too high - It was important to me that I did not miss notifications - Fear of missing notifications would sometimes stress me - Self-reflection is confronting (dealing with uncomfortable emotions) - Self-reflection is difficult (how to correctly identify emotions) **DATA FEEDBACK SESSION** - It was interesting to look at my data | **FUTURE USE**  - I don't find it likely that I will use the tool again, but I won't completely rule it out. |
| **CLIENT**  **ID: 0502**  19 years old  Male  Self-reported diagnosis: none | **EXPERIENCE** - No experience with health apps **EXPECTATIONS** - Was hesitating to participate, because I expected it would be burdensome **MOTIVATION** - I wanted to help research - I was curious to examine patterns in my mood | **SELF-MONITORING** - Notifications were sometimes disturbing - I had to remember my phone and put it on sound - The number of notifications was too high - Difficult to score emotions on a scale - Self-reflection was confronting (being confronted with negative emotions) - Self-reflection would enforce negative emotions **DATA FEEDBACK SESSION** - I was impressed by the data visualizations - Interesting and nice to see my data | **FUTURE USE**  - I would only use the tool again to help research, provided that assessment frequency was lowered |
| **CLIENT**  **ID: 0503**  20 years old  Female  Self-reported diagnose: eating disorder | **EXPERIENCE** - No experience with self-monitoring apps. **MOTIVATION** - I hoped to gain more insight into my mental health. | **SELF-MONITORING** - Assessment frequency was high, but okay for one week. - I sometimes missed notifications because I forgot my phone. - When I was not feeling well I didn't respond to the notifications. - I think I responded to almost all notifications. - It is difficult to score emotions on a scale. - Identifying and labeling emotions is difficult. **DATA FEEDBACK SESSION** - My therapist had difficulties operating the dashboard, so we didn't go into details with the data. | **FUTURE USE**  - I might use the tool again, but it is tiring to do for a long time. - I prefer using the tool as a part of therapy, but I would like to have access to the data myself. |
| **CLIENT**  **ID: 0506**  *(Age unknown)*  Female  Self-reported diagnosis: none | **EXPERIENCE** - I used apps for psychomotor therapy and relaxation. - I am currently using mental health apps. **MOTIVATION** - I thought it might help myself and others. | **SELF-MONITORING** - Notifications were sometimes disturbing. - Assessment frequency was okay. - I tried to respond to as many notifications as possible. | **FUTURE USE**  - I might use the tool again if it was offered to me. |
| **CLINICIAN**  **ID: 0600**  50 years old  Female  Psychologist | **EXPERIENCE** - No experience with digital self-monitoring. - I have used analog self-monitoring techniques in therapy. **EXPECTATIONS** - Better to use than analog self-monitoring methods. **MOTIVATION** - Digital tools are used more and more. - We need to offer clients tools that can help them achieve their goals. | **START-UP SESSION** - It takes time and effort to learn to use the tool. - I didn't personalize my client questionnaire, I wanted to start with the basics.  **SELF-MONITORING** - I monitored my clients' responses and contacted them if I could see they weren't responding.  **DATA FEEDBACK SESSION** - There was a lot of data, I started with simpler visualizations and gradually added things. - If I didn't know the client beforehand, I would not be able to make sense of the data. | **ADDED VALUE**  - The tool allowed us to go more in-depth with problems that we already knew where there. - The tool provided more details and an overview. - We used to tool to identify what was important for the client.  **FUTURE USE** - I will certainly consider using this tool again with my clients. |
| **CLIENT**  **ID: 0601**  50 years old  Female  Self-reported diagnosis: depression | **EXPERIENCE** - No experience with health apps. **EXPECTATIONS** - No specific expectations. - I'm generally skeptical about health apps. **MOTIVATION** - I wanted to help research. | **START-UP SESSION** - I wasn't sufficiently briefed about the assessments. **SELF-MONITORING** - I missed notifications because I was busy. - I missed notifications because I go to bed early. - I had to remember to bring my phone with me and connect it to 4G. - I didn't consider it a problem that I missed some notifications since there was a lot. - I responded to as many notifications as I could, but I missed a lot. - Sometimes difficult to assess whether your mood changed and how much. **DATA FEEDBACK SESSION** - My therapist was able to do a lot with the data, despite low compliance. - I was amazed by what came out of the data. | **ADDED VALUE**  - Useful information came out of the data.  - The tool allows you to get to know yourself better.  **FUTURE USE** - I would be interested in using the tool again. - I would like access to the data myself. |
| **CLIENT**  **ID: 0602**  41 years old  Female  Self-reported diagnosis: Anxiety, eating disorder, OCD, personality disorder | **EXPERIENCE** - I've previously used an app for breathing exercises. - I've previously done analog registration of my emotions, as a part of therapy. **EXPECTATIONS** - I've never been that much into apps. - I thought I might learn something new. | **START-UP SESSION** - The onboarding briefing was insufficient. **SELF-MONITORING** - I would have linked open answer options that allowed me details. - Notifications could be disturbing sometimes. - I would prefer to receive fewer notifications. **DATA FEEDBACK SESSION** - I was surprised how much my therapist could get out of the data. - I needed help interpreting the data, I was not able to do it myself. | **ADDED VALUE**  - It was interesting and useful what came out of the data.  **FUTURE USE** - I would like to use the tool again, but with fewer notifications. - I prefer using the tool as a part of therapy. - I would like to use the tool to monitor my own progress. |
| **CLINICIAN**  **ID: 0700**  44 years old  Male  Psychologist | **EXPERIENCE** - I previously tried using online mental health platforms. - I have worked with analog self-registration in therapy. **EXPECTATIONS**  - Easier to use than analog self-monitoring tools. - Can enable us to provide better care. | **START-UP SESSION** - Dashboard was difficult to use. - It requires good familiarization to use the tool to its full potential. -The more you can personalize the tool the better clients will respond to it. **DATA FEEDBACK SESSION** - There were a lot of visualizations, I didn't make use of them all. - I used the tool to correct my clients' negative self-perceptions of how they were doing. | **ADDED VALUE**  - The tool is interesting and useful.  **FUTURE USE** - I will consider using the tool again with my client. - It would be interesting to use the tool to monitor clients' progress. |
| **CLINICIAN**  **ID: 0800**  41 years old  Female  Nurse | **EXPERIENCE** - I previously tried using online mental health platforms. - I have worked with analog self-registration in therapy. **EXPECTATIONS**  - Easier to use than analog self-monitoring tools. - Will be difficult to use for people with little technical skills and literacy. **MOTIVATION** - Digital mental health tools are useful and we must learn how to use them. | **START-UP SESSION** - It requires a lot of technical skills to use the tool. - I needed a lot of help from others to use the tool. - We practiced using the tool among colleagues. - Personalization required a lot of effort. **DATA FEEDBACK SESSION** - Data was difficult to interpret. - It is not nice to use the tool with a client when you do not fully understand it yourself. - There might be things that I overlooked, or misinterpreted. | **ADDED VALUE**  - The tool provides more reliable data than retrospective accounts. - The tool provides a good way of mapping and discussing clients' problems.  **FUTURE USE** - I hope we will be able to use tools like this more in the future, although it requires a lot of technical skills. |
| **CLIENT**  **ID: 0802**  45 years old  Male  Self-reported diagnosis: Anxiety, bipolar disorder, depression, OCD, psychosis, addiction | **EXPERIENCE** - I use analog methods to keep track of my mental health. - No experience with health apps. **EXPECTATIONS** - Digital tools allow you to do more than analog tools. **MOTIVATION** - I thought it would be interesting to try. | **SELF-MONITORING** - I sometimes missed notifications because I was working. - I sometimes missed notifications because I was sleeping. - The number of notifications was okay. **DATA FEEDBACK SESSION** - The tool made it easier to recollect what happened. | **FUTURE USE** - I might consider using the tool again. |
| **CLINICIAN**  **ID: 1000**  49 years old  Female  Nurse | **EXPERIENCE** - I use analog methods to keep track of my mental health. - No experience with health apps. **EXPECTATIONS** - I was eager to try the tool **MOTIVATION** - Digital technologies have become a big part of our lives.  - It makes sense to use mHealth in mental health care | **START-UP SESSION**  - The tool was not intuitive to use, I constantly felt I was doing something wrong.  - I needed help to use the tool  - I didn’t have enough time to familiarize myself with the tool  **SELF-MONITORING**  - I monitored my clients’ responses and considered contacting my clients’ when I saw they were not responding to the notifications  **DATA FEEDBACK SESSION**  - I struggled to interpret my client’s data  - It was difficult to use the tool with my client because I did not master it fully myself. | **FUTURE USE**  - I might use the tool again if it becomes easier to use |
| **CLIENT**  **ID: 1001**  45 years old  Female  Self-reported diagnosis: ADHD, anxiety, depression, eating disorder, personality disorder, psychosis, addiction, burn-out | **EXPERIENCE** - I previously used a behavioral therapy app. **MOTIVATION** - Integrating mHealth is meaningful for mental health care. - I was curious. | **SELF-MONITORING**  - Some days I was too busy to respond to notifications. - Notifications could be disturbing. I got annoyed and nervous when I wasn't able to fill in the assessments. - I was constantly aware of whether I got a notification. - I would like to receive less notifications. **DATA FEEDBACK SESSION** - It was difficult for my therapist to work with the tool. - Data interpretation was difficult. | **ADDED VALUE**  - The tool provided interesting details about my mental health. - The tool can help people be more actively engaged in their therapy. - The tool can be used to identify problems and strategies.  **FUTURE USE** - It's not necessary for me to use the tool at this point. |
| **CLINICIAN**  **ID: 1200**  51 years old  Female  Other health profession | **EXPERIENCE**  - I used digital self-monitoring tools before in therapy  **EXPECTATIONS**  - It is easier to use and give better insight into clients’ lives than other methods  - Self-monitoring can be difficult for some clients  **MOTIVATION**  - We are missing tools that can help us evaluate the effects of our treatments | **START-UP SESSION**  - Setting up the tool took a lot of time  **DATA FEEDBACK SESSION**  - The many visualizations were somewhat overwhelming  - It was interesting to look at the data | **ADDED VALUE**  - The tool quickly provides you with an overview of how your client is doing  **FUTURE USE**  - I would like to use the tool again  - The tool should be made less burdensome for clients  - It would be interesting to monitor clients for a longer period to evaluate their progress and the effect of treatments |
| **CLIENT**  **ID: 1201**  25 years old  Female  Self-reported diagnosis: OCD | **EXPERIENCE** - No experience with mental health apps. **EXPECTATIONS** - I thought it would make it easier for my therapist to understand my problems. **MOTIVATION** - There is a need for more mHealth in mental health care. - I thought this might help me or others. | **SELF-MONITORING** - Notifications can be disturbing. - People should be allowed to snooze notifications if they are busy. - On good days it is okay to complete the assessments, but on bad days it's difficult. - There are too many notifications. - I did my best to respond to notifications because I wanted the most possible information. **DATA FEEDBACK SESSION** - My therapist reviewed the data without me and told me about the results. - My therapist identified moments when I was feeling bad and tried to understand these. | **ADDED VALUE**  - The tool can help make you aware of what you need to work on. - The tool makes it easier to monitor effects of treatment.  **FUTURE USE** - I would like to keep using the tool to check how I am doing. |
| **CLIENT**  **ID: 1203**  28 years old  Female  Self-reported diagnosis: ADHD, OCD, other | **EXPERIENCE** - No experience with health apps. **MOTIVATION** - I wanted to help research. - I was curious. | **START-UP SESSION** - The assessments does not make sense without personalized questions. **SELF-MONITORING** - Sometimes I couldn't respond to notifications because I was working. -Sometimes the number of notifications was too much. **DATA-FEEDBACK** | **FUTURE USE**  - I would like to use the tool to monitor the effect of my treatment. - I would like to use the tool to monitor my progress and get a better understanding of my mental health . |
